# Pioglitazone modulates metabolic adaptation and peripheral nerve regeneration after injury

**DOI:** 10.1186/s13024-025-00913-1

**Published:** 2025-12-01

**Authors:** Antonia Seitz, Taylan Özöncü, Daniela Sinske, Claudia Klugmann, Daniel Tews, Pamela Fischer-Posovszky, Bernd Knöll, Sofia Meyer zu Reckendorf

**Affiliations:** 1 Institute of Neurobiochemistry, Ulm University, Albert-Einstein-Allee 11, 89081 Ulm, Germany; 2 Department of Pediatrics and Adolescent Medicine, Ulm University Medical Center, Eythstr. 24, 89075 Ulm, Germany; 3 German Center for Child and Adolescent Health (DZKJ), Partner Site Ulm, Ulm, Germany

**Keywords:** Peripheral nerve injury, Schwann cell, Lipid metabolism, PPARɣ, Pioglitazone, Metabolic adaptation, Remyelination, Functional nerve regeneration

## Abstract

Peripheral nerves have an intrinsic capacity for regeneration after traumatic injury, with Schwann cells (SCs) playing a central role in orchestrating this complex process. Critical components of successful regeneration include SC reprogramming into repair SCs, debris removal and metabolic adaptations. Up to now, there are no pharmacological treatments available in the clinics to improve nerve regeneration. In this study, we investigated peroxisome proliferator-activated receptor gamma (PPARɣ) as a therapeutic target in the context of nerve regeneration, since we previously found this transcription factor to be involved in SC reprograming and metabolic adaptations. Therefore, we used a mouse model of sciatic nerve crush injury and applied the PPARɣ agonist pioglitazone (PIO) in two different treatment paradigms: (i) acutely after injury (0–5 days post injury) and (ii) delayed (5–21 days post injury), thereby addressing different phases of regeneration. Our findings revealed that PIO treatment immediately following nerve injury (first treatment paradigm) disrupted SC transition into the repair phenotype and diminished the physiological inflammatory response. As a consequence, axonal and myelin debris clearance was delayed, ultimately resulting in impaired axonal outgrowth and nerve regeneration. In the second treatment paradigm (PIO administration starting five days after injury) SCs had already acquired the repair phenotype and immune cell infiltration had taken place when PIO administration started. There, PIO promoted axonal regeneration, enhanced remyelination, and improved functional recovery. Importantly, PIO treatment increased mitochondrial content in neurons and SCs. In addition, delayed application of PIO induced lipid metabolism, glycolysis and ATP production in SCs, leading to the assumption that improved metabolic conditions mediate enhanced nerve regeneration in this treatment paradigm. These findings show that depending on the timing of PIO treatment, PPARɣ can serve as a potential therapeutic agent to improve nerve regeneration by promoting key metabolic adaptations.

**Supplementary Information:**

The online version contains supplementary material available at 10.1186/s13024-025-00913-1.

## Background

Peripheral nerve injuries (PNIs) present a significant clinical challenge, often resulting from trauma, neuropathic conditions or surgical complications [1]. These injuries can often cause severe motor and sensory deficits, leading to lifelong disability and reduced quality of life [2, 3]. Although peripheral nerves have some regenerative capacity, the process is often inefficient and incomplete, resulting in persistent functional impairments [4].

Peripheral nerve regeneration is a complex biological process characterized by sequential cellular and molecular events, including Wallerian degeneration during the early degenerative phase (in mice: ~days 1–5 post injury) followed by axonal regrowth and remyelination in the later regenerative phase (in mice: day 5 onwards post injury) [5, 6]. In rodents, Schwann cells (SCs) — the principal glial cells of the peripheral nervous system (PNS) [7] — play a central role in this process [8]. Shortly after injury, SCs undergo a phenotypic shift, dedifferentiating into a repair phenotype, characterized by changes in their transcriptional profile, cessation of myelin production, regained proliferative capacity, and the secretion of growth factors, signaling molecules and inflammatory mediators, all of which create a permissive environment for axonal regeneration [8–10].

In the later stages of regeneration, once repair SCs have supported axonal regrowth and facilitated reinnervation of target tissues, they re-differentiate into myelinating SC, thereby enabling the remyelination of regenerated axons [8]. Thus, there are two phases of SC function during PNS regeneration that could be separately targeted by therapeutical approaches. Furthermore, successful tissue repair is inherently energy-demanding, necessitating critical local metabolic adaptations [11]. Nerve injury, in particular, presents a significant metabolic challenge to SCs, which must coordinate both catabolic and anabolic processes, particularly of the lipid metabolism, to drive nerve degeneration and subsequent regeneration.

Despite advancements in pain management, rehabilitation, and microsurgical techniques, clinical outcomes for peripheral nerve regeneration remain poor [4], highlighting the need to explore new regenerative pathways and develop novel therapeutic strategies to enhance nerve repair.

Recent research has underscored the crucial role of metabolic adaptation in SC reprogramming following peripheral nerve injury [12, 13]. Our previous work revealed that the transition of SCs from a myelinating to a repair phenotype is closely linked to significant alterations in lipid metabolism involving the peroxisome proliferator-activated receptor gamma (PPARɣ) [12]. PPARɣ, a nuclear receptor, regulates gene expression related to lipid metabolism, glucose homeostasis, cell respiration and inflammation [14]. Its activation has been shown to exert pro-regenerative and neuroprotective effects in various neuronal injury models including ischemic stroke [15–17] or spinal cord injury [18–21]. Furthermore, recent research has highlighted the critical role of dynamic metabolic adaptation in repair SCs during nerve regeneration. Nerve de- and regeneration are marked by distinct metabolic shifts, including the early induction of oxidative phosphorylation (OxPhos) and catabolic autophagy, followed by late anabolic processes such as lipid synthesis required for remyelination [13].

In this study, we evaluate PPARɣ as a potential therapeutic target in a peripheral nerve injury model using the PPARɣ agonist pioglitazone (PIO). PIO, a thiazolidinedione-class drug, is primarily used to treat type 2 diabetes due to its insulin-sensitizing properties [22]. Beyond its systemic metabolic effects, PIO has demonstrated neuroprotective benefits in multiple models of neurological disorders, including neurodegenerative diseases, brain and spinal cord injury [19, 23–28]. There, PIO seemed to elicit functions on mitochondria biogenesis and respiration, thereby contributing to neuroprotection [24, 25]. However, the precise molecular mechanisms underlying the observed beneficial effects remain poorly understood in the context of nerve regeneration therapy.

Here, we demonstrate that acute PIO treatment following nerve crush impairs SCs from transitioning into the repair phenotype, thereby hindering nerve regeneration. In contrast, initiating PIO treatment after the SC switch enhances lipid metabolism, mitochondrial abundance, and ATP production in SCs. Additionally, PIO increases mitochondrial abundance in regenerating axons. These metabolic adaptations appear to promote axonal outgrowth, remyelination, and overall functional recovery. Thus, we propose PIO as a potential therapeutic agent to improve energy homeostasis in SCs and thereby nerve regeneration after injury.

## Methods

### Primary Schwann cell culture

Wild-type C57BL/6J mice, aged 12–16 weeks and of both sexes, were euthanized using CO_2_ followed by cervical dislocation. Sciatic nerves from both hind limbs were dissected and transferred into DMEM (Gibco) supplemented with 10% FBS (Sigma) and 1% Pen/Strep (Gibco) and maintained for 3 days, with one medium change during this period. Enzymatic digestion of the nerves was then performed according to the protocol established by Haastert et al. [29], followed by mechanical trituration. The cell suspension was transferred into an ultra-low attachment plate (Corning Costar^®^, 3471) and incubated for 48 h to enrich for SCs. Primary SCs were subsequently seeded onto poly-L-lysine- and laminin-coated coverslips for immunocytochemistry (ICC) or into 12-well plates (Sarstedt) for RNA isolation, using the above-mentioned culture medium. For immunocytochemistry, 3 × 10^4^ cells were seeded per 12 mm diameter glass coverslip, while 3.5 × 10^5^ cells were seeded for RNA isolation. After 24 h of cell attachment, SCs were treated with either 10 µM PIO (Sigma-Aldrich, dissolved in DMSO) or DMSO (vehicle control) for 5 consecutive days. The drug-containing medium was exchanged every second day.

### Primary dorsal root ganglia (DRG) neuron culture

DRGs were isolated from adult C57BL/6J mice, and subjected to enzymatic digestion using collagenase II and dispase II (1 h, 37 °C), followed by mechanical trituration to obtain a single-cell suspension. 3 × 10^3^ dissociated DRG neurons were seeded onto 12 mm diameter glass coverslips pre-coated with poly-L-lysine and laminin. Cultures were maintained in Neurobasal Medium (Gibco) supplemented with 10% FBS (Sigma), 2% B27 supplement (Gibco), 1% L-glutamine (Gibco) and 1% Gentamycin (Gibco). Neurons were allowed to adhere and extend neurites for 24 h prior treatment. DRG cultures were subsequently exposed to either pioglitazone (10 µM) or DMSO (vehicle control) for 5 consecutive days, with medium containing the respective treatment refreshed every other day.

### Mice

Wild-type C57BL/6J mice, aged 8-12-weeks, were acquired from Charles River and bred in-house. Male and female mice have been used and were equally distributed between all groups. The mice were housed in groups of 2–4 animals per cage under controlled environmental conditions, including a 12-hour light/dark cycle, a constant temperature of 22 °C, and 60% humidity. Food and water were provided *ad libitum*. All experimental procedures complied with institutional guidelines and German animal protection laws and were approved by the appropriate regional government authority (Regierungspräsidium, Tübingen, Germany).

### Experimental design

Wild-type C57BL/6J mice were randomly and equally assigned to treatment and control groups. Pioglitazone was administered via oral gavage at a dose of 20 mg/kg body weight. As a vehicle control, an equivalent volume of DMSO was administered under the same conditions. Treatment was administered during two distinct phases following sciatic nerve crush: daily during the early phase (days 0 to 5 post injury, Setup 1) or every other day during the later phase (days 5 to 21 post injury, Setup 2). Molecular analyses were conducted on uninjured control samples and at 3, 5, 10, 14- and 21-days post injury. Functional recovery was assessed at baseline (day − 1), and on days 1, 3, 5, 10, 14, 17- and 21-days post injury.

### Sciatic nerve crush

Mice were anesthetized with 5% isoflurane delivered in oxygen at a flow rate of 3 L/min and administered a subcutaneous injection of carprofen (5 mg/kg body weight) for analgesia. A small incision was made in the skin overlying the upper thigh, and the sciatic nerve was carefully exposed by dissecting the fascial plane between the gluteus maximus, biceps and quadriceps femoris muscles. The nerve was crushed at the mid-femoral level using hemostatic scissors, applied orthogonally two times for 7 s. To mark the injury site, the scissors were dipped in activated charcoal powder prior to the crush. Following the procedure, the skin was sutured with two stiches. Post-operative care included the administration of carprofen (0.07 mg/ml drinking water), beginning one day prior to surgery and continuing for three days post-surgery. The surgical procedure was performed on the right hind limb, with the left hind limb serving as the internal control.

### Ex vivo sciatic nerve samples

Wild-type C57BL/6J mice, aged 12–16 weeks and of both sexes, were euthanized using CO_2_ followed by cervical dislocation. Sciatic nerves from both hind limbs were dissected. One sciatic nerve was immediately frozen to serve as a control (0 h). The contralateral sciatic nerve was placed in tubes containing DMEM supplemented with 10% FBS and 1% Pen/Strep, along with either PIO (10 µM) or DMSO (vehicle control). The tubes were incubated at 37 °C for 48 h.

### Quantitative polymerase chain reaction

Total RNA was isolated from distal murine sciatic nerves using TRIzol reagent (Qiagen) followed by purification with the RNeasy kit (Qiagen), according to the manufacturer’s protocol. On-column DNase digestion with the RNase-free DNase set (Qiagen) was performed for 15 min to eliminate genomic DNA contamination. Reverse transcription was carried out with 1 µg of RNA using a reverse transcription reagent kit (Takara, #RR036A). Quantitative PCR (qPCR) was performed on a LightCycler 96 system (Roche) using TB Green Premix Ex Taq PCR master mix (Takara). The threshold cycle (Ct) values for each sample were determined using LightCycler Software. Gene expression levels were normalized to the housekeeping gene *Hprt* (hypoxanthine-guanine phosphoribosyltransferase 1). Primers are provided in Table 1.

For primary murine SCs, total RNA isolation was performed using the RNeasy kit (Qiagen) according to the manufacturer’s instructions, followed by the same reverse transcription and qPCR procedures as described above.

### Immunohistochemistry

Sciatic nerves were fixed in 4% paraformaldehyde (PFA), embedded in paraffin and sectioned into 5 μm slices using a microtome (Leica). Immunohistochemistry was performed using the following primary antibodies: anti-PPARɣ (rabbit, 1:5000, Abcam, ab59256), anti-PLIN2/ADFP (rabbit, 1:500, Novus biologicals, NB110-40877), anti-CD45 (rat, 1:100, BD Pharmingen, 550539), and anti-cJUN (rabbit, 1:500, Cell Signaling, #9165). Detection was achieved using biotin-conjugated secondary antibodies (1:500, BA-1000, Vectorlabs) and a peroxidase-based ABC kit (PK-6100, Vectorlabs) with DAB as substrate (Vectorlabs, SK-4100). For fluorescence staining, Goat anti-Rabbit IgG (H + L) Cross-Adsorbed Secondary Antibody, Alexa Fluor™ 546 (1:500, Invitrogen, A-11010) was used followed by DAPI counterstain. Nerve samples were mounted using mowiol.

Muscles and hind paw tissue were collected, fixed in 4% PFA, and cryoprotected in 30% sucrose. Tissues were sectioned into 30 μm slices using a cryostat (Leica). NMJs were stained with α-bungarotoxin (BTX, 1:500, Molecular Probes, B35451) alongside with the primary antibody anti-Synaptophysin-1 (SYN1, guinea pig, 1:200, Synaptic System, 101004) overnight at 4 °C. Sections were then incubated with the secondary antibody CF(R) Donkey Anti-Guinea Pig IgG (H + L), Highly Cross-Adsorbed 633 (1:500, Biotium, 20171) for 2 h at room temperature. Hindpaw skin was stained with the primary antibody anti-UCHL1/PGP9.5 (rabbit, 1:200, Proteintech, 14730-1-AP) overnight at 4 °C, followed by incubation with the secondary antibody Goat anti-Rabbit IgG (H + L) Cross-Adsorbed Secondary Antibody, Alexa Fluor™ 546 (1:500, Invitrogen, A-11010). Muscle and hind paw samples were mounted using ProLong Gold Antifade Mountant with DAPI (Thermo Fisher Scientific).

### Immunocytochemistry

Primary murine SCs and DRG neurons were incubated with MitoTracker Red™ CMXROS (Invitrogen, M7512) at a final concentration of 200 nM in serum-free medium for 30 min at 37 °C. Following incubation, cells were fixed with 4% PFA containing 5% sucrose prior to immunostaining. The following primary antibodies were used at 4 °C over night: anti-S100β (mouse, 1:1000, Invitrogen, MA5 29546), and anti-βIIITUB (mouse, 1:2000, BioLegend, 801201). Subsequently, samples were incubated with secondary antibody for 2 h at room temperature: Goat anti-Mouse IgG (H + L) Cross-Adsorbed Secondary Antibody, Alexa Fluor™ 488 (1:500, Invitrogen, A-11001). Schwann cells and DRG neurons were mounted using ProLong Gold Antifade Mountant with DAPI (Thermo Fisher Scientific).

### Imaging and quantification

Primary SCs and DRG neurons were imaged using a Zeiss Axio Observer fluorescence microscope with a 63x oil immersion objective. Mitochondrial analyses were performed on 30 individual SCs, and on 30 neuronal cell bodies and 30 neurites per sample. SCs, neuronal somata, and neurites were manually outlined using the ‘segmented line’ tool in ImageJ. A functional color threshold was applied to standardize brightness levels and to distinguish specific mitochondrial staining from background signal. In primary SCs, the MitoTracker^+^ area (relative to the S100β signal) was quantified using the automated ‘analyze particles’ function in ImageJ. For neuronal soma analysis, the number of MitoTracker^+^ punctae was determined using the ‘find maxima’ function in ImageJ. In neurites, colocalization of MitoTracker and βIIITUB signals was assessed using the ‘selection and overlay analysis’ tool. Neurite length was measured using the ‘measure’ function in ImageJ.

Histological nerve sections were imaged using a Keyence BZ-X810 fluorescence microscope with a 20x objective. One section per animal, either from uninjured nerves or ~ 5 mm distal to the lesion site, was analyzed. Each nerve area was outlined using the ‘segmented line’ tool, and a functional color threshold was applied. The positive area (PLIN2) or the number of stained objects (CD45, cJUN) was quantified using the automated ‘analyze particles’ function. The area fraction of PPARɣ^+^ signals (brown) in nerve sections was quantified using ImageJ’s ‘color deconvolution’ application, which selectively isolates and analyzes specific staining signals.

Hind paws and muscles were imaged using a Keyence BZ-X810 fluorescence microscope with a Z-stack function (1 μm pitch). Two sections per hind paw and muscle sample were quantified in ImageJ, and the mean value of both sections was used for statistical analysis. For NMJ quantification, a minimum of 35 synapses per animal were analyzed. Each synapse was outlined using the ‘segmented line’ tool and the colocalization of BTX and SYN1 signals was assessed using the ‘colocalization analysis’ tool. The epidermal region of hind paws was encircled using the ‘segmented line’ tool, and the PGP9.5 positive area was quantified using the automated ‘analyzed particles’ function in ImageJ after applying a functional color threshold.

### Seahorse mitostress assay

SC cultures were prepared and 4 × 10^4^ primary SCs were seeded into each well of a Seahorse XF Microplate (Agilent Technologies) pre-coated with poly-L-lysine and laminin. Primary SCs were treated for 5 consecutive days with either PIO (10 µM) or DMSO (vehicle control) prior to conducting the Mitostress assay. Before measurement, cells were washed twice with Seahorse XF DMEM medium supplemented with 10 mM glucose, 1 mM pyruvate and 1 mM glutamine, followed by a 1 h incubation at 37 °C in a non-CO_2_ incubator to equilibrate. Oxygen consumption rates (OCR) and extracellular acidification rates (ECAR) were measured using a Seahorse XFe96 Flux –Analyzer (Agilent Technologies). Mitochondrial function was assessed by sequentially injecting 1 µM oligomycin to determine ATP-linked respiration, 2 µM carbonyl cyanide-*p*-trifluoromethoxyphenylhydrazone (FCCP) to determine maximal substrate oxidation capacity, and 0.5 µM antimycin A plus 0.5 µM rotenone to quantify non-mitochondrial respiration. Optimal concentrations of these compounds were titrated beforehand. Mitochondrial ATP and glycolytic ATP production rates were calculated according to Desousa et al. [30] and the obtained data were normalized using Janus Green incorporation as a proxy for total cell number.

### Electron microscopy

Uninjured control sciatic nerves and injured nerves, ~ 8 mm distal to the lesion site, were fixed overnight in 4% paraformaldehyde (PFA) and subsequently post-fixed in 2.5% glutaraldehyde for 24 h before being embedded in epoxy resin. Ultrathin Sect. (80 nm) were prepared from the region of interest. For each timepoint three to four murine nerves were analyzed. For the quantification of myelin phenotype and axonal parameters, three randomly selected frames per nerve at each timepoint were imaged at 2,500x magnification, and the entire area (3200 µm^2^) of each image was analyzed. To assess remyelination following nerve injury, 25 arbitrary axons per frame were analyzed for uninjured controls and at 21 days post injury. For the 10- and 14- day post injury timepoints, all remyelinated axons within each frame were quantified, and the mean values were calculated per sample. To determine the myelin G-ratio at 21 dpi, 75 randomly selected axons per mouse were analyzed at 2,500x magnification. The G-ratio was calculated using following formula:

$$\:G-ratio=\frac{{R}_{I}}{{R}_{O}}$$

where R_I_ represents the inner (axonal) radius and R_O_ the outer (myelinated fiber) radius. To assess mitochondrial abundance in axons and SCs, a minimum of 20 axons and SCs per nerve at each timepoint were imaged at 12,000x magnification. SC cytoplasm, axons, and individual mitochondria were manually outlined, and their area and abundance were measured.

### Bulk RNA sequencing

RNA was isolated as described above. RNA concentration, purity and integrity were assessed, and only samples with an RNA integrity Number (RIN) greater than 7.0 were selected for downstream analysis. Library preparation and sequencing were performed by Novogene Co. using the Illumina NovaSeq platform, employing a paired-end 150 bp sequencing strategy. Graphs were generated using SRPlot platform (https://www.bioinformatics.com.cn/srplot).

### Behavioral assessment

Behavioral assessments were conducted on both uninjured and injured hind paws at baseline (day − 1) and on days 1, 3, 5, 10, 14, 17- and 21-days post injury to capture various stages of nerve healing and regeneration. All investigators performing behavioral assessments were blind to the treatment conditions. Mice were acclimatized to the testing environment for 1 h prior testing.

Mechanical sensitivity was assessed using the pinprick test. Mice were placed on an elevated wire grid, allowing access to the planar surface of the hind paws. A pinprick needle (26G) was applied to the central plantar surface of the hind paw with sufficient force to indent but not penetrate the skin. A rapid hind paw withdrawal was considered as a positive response. Each hind paw (uninjured and injured) was tested three times and the cumulative score was evaluated for each paw at each time point.

Mouse gait was assessed pre- and post-injury using the CatWalk XT system (Noldus Information Technology). Mice were allowed to freely traverse a 30 cm long glass corridor on a glass plate, and illuminated paw prints were captured with a high-speed camera. The following settings were applied consistently across all animals and timepoints: maximum speed variation of 200%, speed range of 1.0 to 100.0 cm/second, and a minimum of 12 consecutive steps per run. A minimum of 3–6 compliant runs were recorded per session. Following image acquisition, each video was manually reviewed to correct any misclassified paw placement not accurately identified by the system’s automated algorithm. Run parameters were averaged for each mouse. In this study, the analysis focused on the right (injured) and left (uninjured) hind paws of each mouse. The intermediate toe spread was manually assessed by measuring the distance between the second and forth toe of the left and right hind paws. The sciatic functional index (SFI) was calculated using following the formula [31]:

$$\begin{aligned}SFI=\text{118,9} *\left(\frac{{TS}_{E}-{TS}_{N}}{{TS}_{N}}\right)-\text{51,2}\cr  *\left(\frac{{PL}_{E}-{PL}_{N}}{{PL}_{N}}\right)-\text{7,5}\end{aligned}$$

Where TS represents the toe spread and PL the print length, and the subscripts E and N indicate the Experimental (right) and Normal contralateral (left) hind paws, respectively.

The grip test was employed to evaluate motor function and grip performance in mice after nerve injury. Mice were placed on top of an elevated wire grid and allowed to grab the wires. The grid was then inverted, and the mouse filmed for one minute. The number and quality of grips were subsequently assessed over a 15-second period of constant movement. Grip performance was scored as follows: Score 1: loose grip using only the toes of the hind paw. Score 2: firm grip using the entire hind paw (see also Fig. 4s). Scores 2 for each mouse were summed up across each trial at each timepoint and normalized to the uninjured hind paw.

### Statistical analysis

A minimum of three biological replicates were analyzed for all experiments. The number of independent samples (*n*) is indicated in the figure bars or figure legends. Statistical analysis and graph generation were performed using GraphPad Prism software (GraphPad Software, Inc.). Outliers were identified using the ROUT method. Sample groups were tested for Gaussian distribution using the D’Agostino-Pearson omnibus normality test. As some groups were not normally distributed or had insufficient sample sizes (*n* < 10) for normality testing, the nonparametric unpaired two-sided Mann-Whitney test was used to calculate significance unless otherwise stated. Statistical significance is denoted as *P ≤ 0.05, **P ≤ 0.01, ***P ≤ 0.001, and ****P ≤ 0.0001. Data are presented as mean with standard deviation (SD) unless otherwise specified.

## Results

### Schwann cells respond to PIO treatment in a PPARγ dependent manner in vitro

**Fig. 1:**
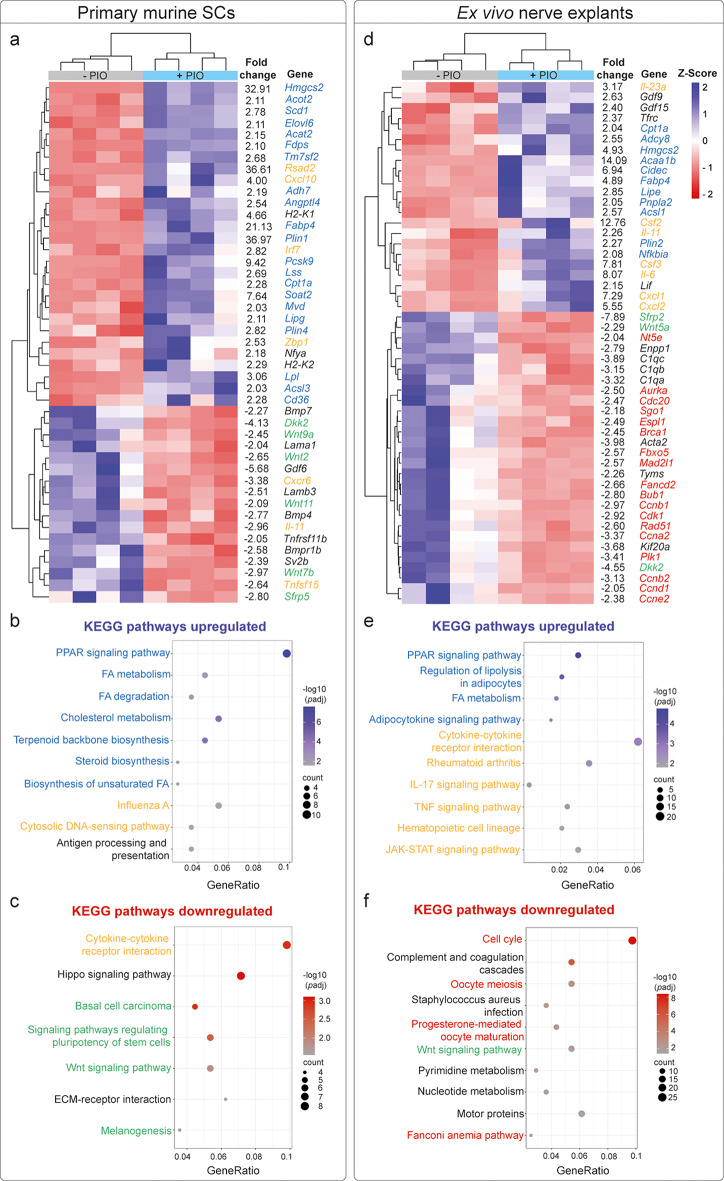
RNA sequencing analysis reveals PIO-induced gene expression changes in primary murine Schwann cells and sciatic nerve explants. **a**-**c**: Bulk RNA sequencing of primary murine SCs treated with either PIO (+ PIO) or DMSO (- PIO) for 48 h. **a**: Heat map of the most significantly differentially expressed genes involved in the KEGG pathways in (**b**) and (**c**). b, c: Bubble plots of the top ten significantly upregulated (**b**) or top seven downregulated (**c**) KEGG pathways associated with differentially expressed genes in primary SCs treated with PIO vs. DMSO. **d**-**f**: Bulk RNA sequencing of sciatic nerve explants from uninjured control nerves or ex vivo injured nerves treated with either PIO (- PIO) or DMSO (- PIO) for 48 h. **d**: Heat map of the most significantly differentially expressed genes involved in the KEGG pathways in (**e**) and (**f**). **e**, **f**: Bubble plots of the top ten significantly upregulated (**e**) or downregulated (**f**) KEGG pathways associated with differentially expressed genes in e*x vivo* injured nerve explants treated with PIO vs. DMSO. *n* = 4 biological replicates for primary murine SCs and ex vivo sciatic nerve explants. FA, fatty acid; KEGG, Kyoto Encyclopedia of Genes and Genomes; PIO, Pioglitazone; SC, Schwann cell

First, we aimed to investigate whether SCs respond to PIO treatment by adapting PPARγ dependent pathways (e.g. lipid metabolism, inflammation, cell cycle) as previously published for different cell types [12, 32, 33]. Therefore, we isolated primary SCs, treated them with PIO for 48 h and analyzed gene expression by bulk RNA sequencing (Fig. 1a-c, Suppl. Figure 1a-b). Here, we confirmed that PIO changes gene expression in SCs, resulting in different clustering of control (- PIO) and PIO (+ PIO) treated cells and in the upregulation of 102 vs. downregulation of 112 genes (Suppl. Figure 1a-b). As previously reported, PIO treatment strongly activated genes related to lipid metabolism (Fig. 1a, light blue), while genes related to Wnt signaling were downregulated (Fig. 1a, green) [34, 35]. Interestingly, genes associated with inflammatory response were partially induced and partially repressed (Fig. 1a, yellow). Those changes were also reflected in the KEGG pathway analysis with strongest upregulated pathway being PPAR signaling (Fig. 1b-c). Taken together, our analysis of PIO treated SCs in culture confirmed the expected PPARγ specific response (Fig. 1a-c).

Since cultured SCs are per default in the repair SC state, it is not possible to analyze the effects of PIO in combination with an injury response. In order to overcome this obstacle, we employed our previously established ex vivo nerve injury model [12]. Therefore, we isolated murine sciatic nerves and snap froze them immediately (representing the uninjured nerves) or after an incubation time of 48 h in media with either control (- PIO) or PIO (+ PIO) treatment. Thereafter, bulk RNA sequencing was performed to analyze PIO effects in the context of nerve injury (Fig. 1d-f, Suppl. Figure 1c-d). This confirmed the transcriptional injury response in nerve explants, as well as the adapted injury response in PIO treated explants (Suppl. Figure 1c-d). In line with previous observations, lipid metabolism associated genes were strongly induced (Fig. 1d, light blue), which correlated to PPAR signaling pathway as most significant upregulated KEGG pathway (Fig. 1e). In this experimental setup, which included additional cell types beyond SCs, inflammation associated genes were activated by PIO treatment in combination with the injury response (Fig. 1d, yellow and 1e). This was in contrast to the pure SC cultures, where PIO treatment had mixed effects on inflammatory response (Fig. 1a-c). In addition, PIO treatment resulted in a strong inhibition of cell cycle related genes in combination with the injury response (Fig. 1d, red and 1f), which is in line with previous reports about PIO-mediated cell cycle suppression in different cell types [32, 36, 37].

Finally, we analyzed pathways commonly regulated in SCs and nerve explants in order to find out whether some PIO mediated responses can occur independent of the differentiation state of the SCs (Suppl. Figure 2). Interestingly, only a small subset of genes seemed to be commonly regulated (7 genes up, 20 genes down; Suppl. Figure 2a, d) indicating that most SC responses strongly depend on the differentiation state of SCs. Nevertheless, analyzing the commonly upregulated genes pinpointed at fatty acid metabolism, PPAR pathway and mitochondria inner membrane as the most robustly altered GO terms after PIO treatment (Suppl. Figure 2b). Commonly downregulated genes on the other hand belonged to GO terms associated with extracellular matrix modification or cell-cell-signaling (Suppl. Figure 2c), which could be attributed to PIOs capacity of shifting SCs to the myelinating rather than the repair phenotype.

In sum, PIO treatment of SCs showed expected effects on lipid metabolism, inflammation and cell cycle regulation, while no off-target effects could be detected on transcriptional level.

### PIO treatment elicits PPARγ dependent effects on injured nerves in vivo

**Fig. 2:**
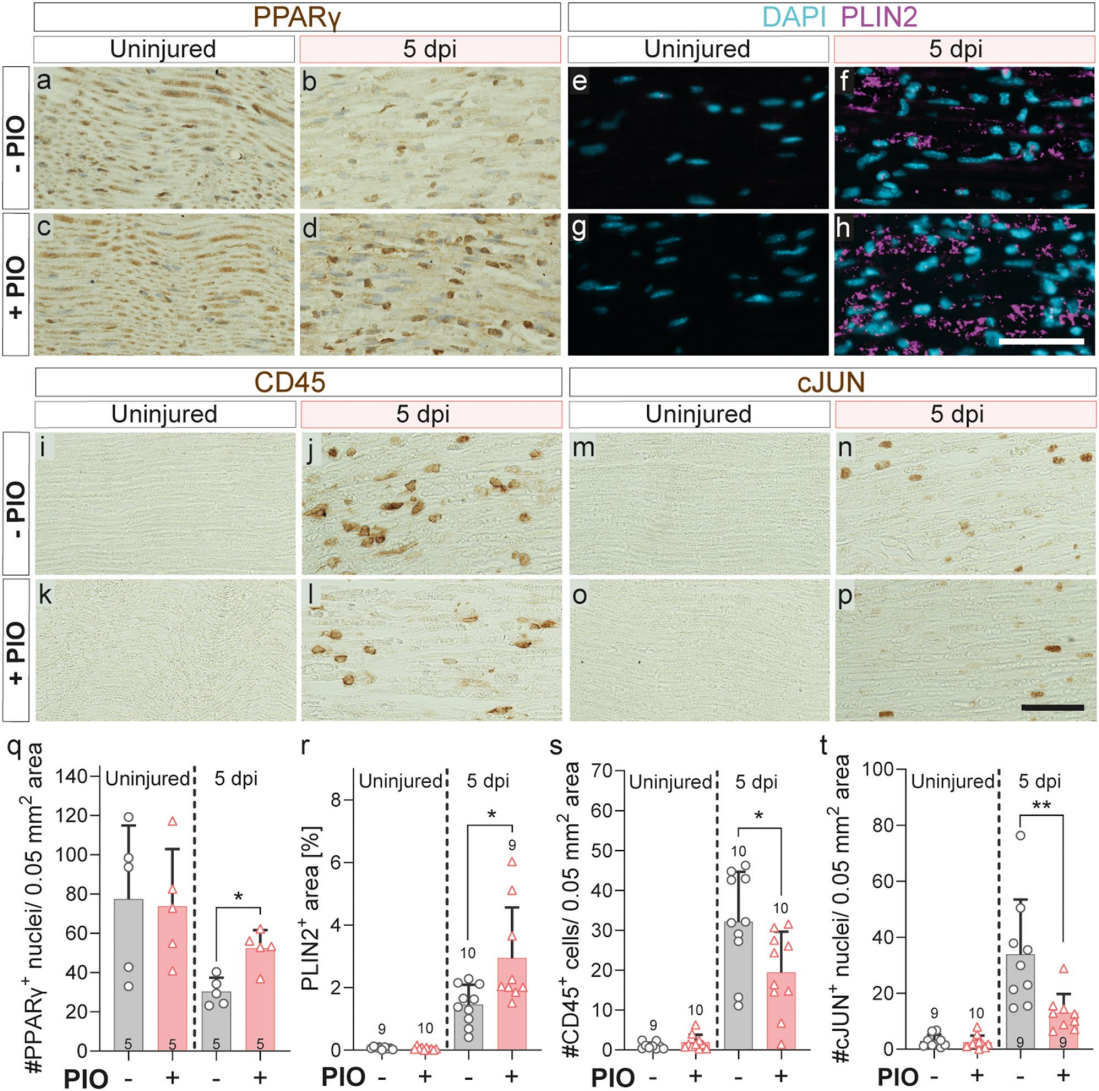
Systemic PIO treatment immediately following nerve crush elicits PPARɣ dependent effects in injured nerves. **a**-**p**: Histological analyses of longitudinal sections of control or injured murine sciatic nerves at 5 days post injury. Images of injured nerves were taken distally to the lesion site. Mice were treated for 5 consecutive days with either PIO (+ PIO) or DMSO (- PIO; vehicle control), starting on the day of injury. Sections were stained for PPARγ (**a**-**d**), PLIN2 (marker for lipid droplets; **e**-**h**), CD45 (marker for immune cells; **i**-**l**) and cJUN (marker for repair SCs; **m**-**p**). **q**-**t**: Quantification of staining at 5 dpi and in uninjured nerves comparing – PIO vs. + PIO treatment: **q**: PPARɣ-positive nuclei, *n* = 5. **r**: PLIN2-positive area, *n* = 9–10. **s**: number of CD45-positive immune cells, *n* = 9–10. **t**: number of cJUN-positive repair SCs, *n* = 9–10. Each dot represents a single mouse. Statistical significance was determined using a two-sided Mann–Whitney test. Error bars represent mean with SD. Significance levels are indicated by asterisks (*P ≤ 0.05, **P ≤ 0.005, ***P ≤ 0.001). Scale bar in (**h**) and (**p**): 50 μm (applies to all images (**a**-**p**)). DAPI, 4′,6-Diamidin-2-phenylindol; dpi, days post injury; PIO, Pioglitazone; PLIN2, Perilipin 2; SC, Schwann cell

Since PIO altered gene expression in SCs *in vitro/ex vivo*, we went on to analyze its effects on injured sciatic nerves in vivo. Therefore, we used the sciatic nerve crush model in mice, and combined it with a systemic PIO or control treatment applied daily from the day of surgery until 5 days after. Histological analysis of nerves at 5 days post injury (dpi) revealed a downregulation of PPARγ after injury (Fig. 2a-b, q), which is in line with our previous observation ex vivo [12]. PIO treatment blocked this downregulation with PPARγ levels to a strong degree (Fig. 2a-d, q). Next, we wanted to investigate, if PIO mediated PPARγ upregulation in injured nerves, also resulted in adaptations of the lipid metabolism, as we had previously seen in the *in vitro/ex vivo* approach (Fig. 1). Therefore, we stained the lipid droplet (LD) coating protein PLIN2 (perilipin 2) and found an accumulation of LDs in injured nerves (Fig. 2e-f, r). In line with our expectations, LD accumulation as indicated by PLIN2 staining was increased in PIO treated injured nerves (Fig. 2g-h, r).

Inflammatory response in terms of pro-inflammatory gene expression was contradictory in our in vitro and ex vivo systems (Fig. 1). Hence, in order to define PIO mediated effects on the actual inflammatory response on cellular level in vivo, we analyzed immune cell infiltration in injured nerves using CD45 staining. Here, we saw that while injury resulted in a massive immune cell infiltration in control treated mice, this response was blunted as a result to PIO treatment (Fig. 2i-l, s). This was in line with previous reports on inflammation in PIO treated injured nerves [38].

Since repair SCs regain proliferative capacity and PIO treatment inhibited cell cycle related genes in our ex vivo experiments (Fig. 1d, f), we investigated whether PIO treatment can affect the SC reprogramming from myelinating to repair SCs in vivo. In order to analyze this, we stained the prototypical repair SC marker cJUN and found a strong upregulation in control treated injured nerves (Fig. 2m-n, t) in line with the literature [8]. PIO treatment blocked the SC reprogramming (Fig. 2p, t), which is in line with previous observations in our group in an ex vivo model [12].

Next, we wanted to assess whether in addition to SCs also infiltrating immune cells might contribute to the detected PLIN2 and cJUN signals. Hence, we co-stained lymphocytes and macrophages using α-CD45 and α-F4/80 together with either PLIN2 or cJUN (Suppl. Figure 3). Here we only found a negligible number of immune cells to be PLIN2^+^ or cJUN^+^, leading to the assumption that SCs are the predominant cell type expressing cJUN and PLIN2 at 5 dpi (Suppl. Figure 3).

Taken together, in vivo systemic PIO application in mice with a sciatic nerve injury recapitulates SC responses demonstrated *in vitro/ex vivo* by modifying inflammatory response, enhancing lipid production/accumulation and interfering with SC reprogramming. These results position PIO as a promising therapeutic agent for regulating SC responses in injured nerves.

### PIO alters debris clearance, axonal regeneration and remyelination in a time dependent manner

So far, our results demonstrated that nerves/SCs treated with PIO activate pathways (e.g. lipid metabolism) that rather favor the lipid synthesis dependent myelinating SC phenotype, while pathways favoring repair SCs (e.g. cJUN expression, cell cycle, inflammation) are blocked. This led us to speculate, that PIO treatment in the acute phase after injury would most likely block the physiological injury response, resulting in impaired regeneration. In contrast, PIO treatment in a later phase, after myelinating SCs have reprogrammed to repair SCs might improve regeneration by promoting the remyelination of injured nerves.

**Fig. 3:**
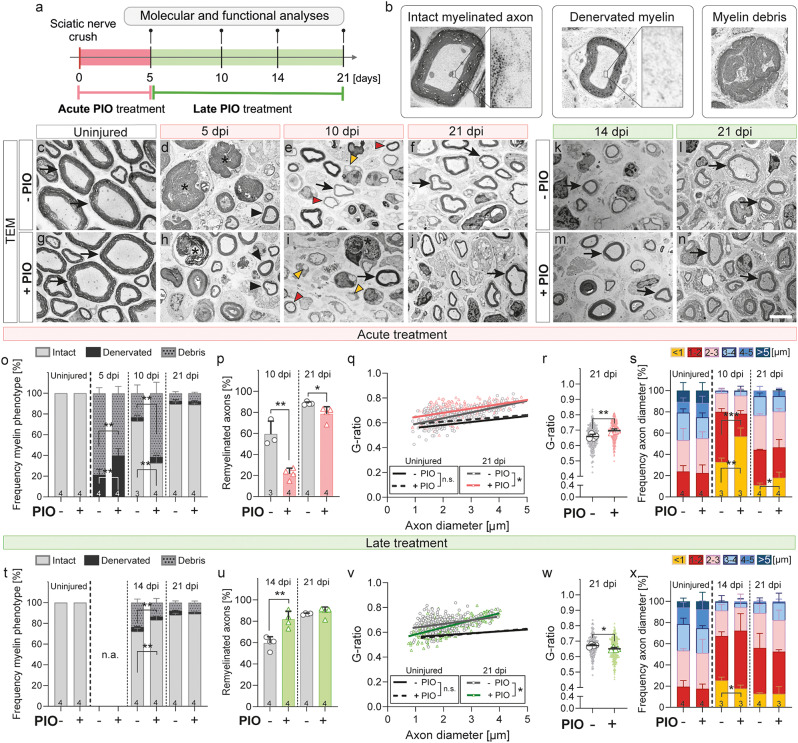
PIO treatment following nerve injury alters debris clearance, axonal regeneration and remyelination. **a**: Schematic representation of the experimental design, including “acute” (days 0–5 post injury, daily) and “late” (days 5–21 post injury, every second day) PIO treatment after nerve injury. **b**: Magnification of Transmission electron microscopy (TEM) images of intact myelinated axons, denervated myelin, and myelin debris. Inserts show higher-magnification views of the axonal compartment, highlighting the presence of tubulin structures within intact myelinated axons and their absence in denervated myelin sheaths. **c**-**n**: TEM images of murine sciatic nerve cross-sections. 5, 10, 14, and 21 days post injury (dpi) and in uninjured controls. Black arrows indicate intact myelinated axons, arrowheads show denervated myelin, and asterisks mark myelin and axonal debris as defined in (**b**). Orange arrowheads highlight regenerating axons with a diameter < 1 μm, while red arrowheads indicate regenerating axons with diameters between 1–2 μm. **o**: Quantification of the percentage of intact myelin sheaths, denervated myelin and myelin debris in sciatic nerves at 5, 10, and 21 dpi and in uninjured controls, in mice with “acute” PIO treatment. **p**: Quantification of the percentage of remyelinated axons in sciatic nerves at 10 and 21 dpi in mice with “acute” PIO treatment. **q**: Analysis of myelin sheath thickness by calculating the myelin G-ratio, plotted against axon diameter, in uninjured control nerves and at 21 dpi in mice receiving “acute” PIO treatment. **r**: Distribution of individual G-ratio values at 21 dpi in mice with “acute” PIO treatment. **s**: Distribution of axon diameters in sciatic nerves at 10 and 21 dpi, and in uninjured controls in mice with “acute“ PIO treatment. **t**: Quantification of the percentage of intact myelin sheaths, denervated myelin and myelin debris in sciatic nerves at 14 and 21 dpi and in uninjured controls in mice with “late” PIO treatment. **u**: Quantification of the percentage of remyelinated axons in sciatic nerves at 14 and 21 dpi in mice with “late“ PIO treatment. **v**: Analysis of myelin sheath thickness by calculating the myelin G-ratio, plotted against axon diameter, in uninjured control nerves and at 21 dpi in mice with “late” PIO treatment. **w**: Distribution of individual G-ratio values at 21 dpi in mice receiving “late” PIO treatment. **x**: Distribution of axon diameters in sciatic nerves at 14 and 21 dpi, and in uninjured controls in mice with “late“ PIO treatment. Bars in (**o**, **s**, **t**, **x**) represent the mean of 3–4 individual mice each. Each large dot in (**r**, **w**) represents the mean value of one biological replicate, and small background dots indicate individual G-ratio measurements. Statistical significance was calculated using an unpaired T-test. All error bars show mean with SD. Statistical significance is indicated by asterisks (*P ≤ 0.05, **P ≤ 0.005, ***P ≤ 0.001). Scale bar in (**n**) is 5 µm and applies to images (**c**-**n**). Dpi, days post injury; n.a., not applicable; PIO, Pioglitazone; TEM, transmission electron microscopy

In order to test this hypothesis, we used again the sciatic nerve crush model and employed two different treatment protocols: (i) “acute” PIO treatment included – as described above – daily PIO treatments, starting on the day of surgery for 5 consecutive days and (ii) “late” PIO treatment, including PIO treatment every second day, starting on 5 dpi until sacrifice, latest 21 dpi; see Fig. 3a). In order to test the efficiency of the PIO treatment in the two treatment paradigms, we analyzed *Pparγ* mRNA expression at the end of each treatment (5 dpi for acute treatment, 21 dpi for late treatment). As expected, we found elevated *Pparγ* expression for both treatments, although not significant in the late treatment (Suppl. Figure 4a, i). Histology of nerves from mice receiving the acute treatment showed that the injury mediated decrease of PPARγ was blunted at 5 dpi on protein level (Suppl. Figure 4h; as already shown in Fig. 2a-d, q). Interestingly, PPARγ gradually decreased in PIO treated mice after PIO was withdrawn, finally even reaching lower PPARγ protein levels than control-treated mice at 21 dpi (Suppl. Figure 4f-h). This indicates that SCs of injured nerves finally do downregulate PPARγ after PIO treatment is withdrawn (Suppl. Figure 4f-h). On the other hand, histological analysis of nerves from mice that had received late PIO treatment, showed that PPARγ protein levels were significantly elevated at 14 and 21 dpi (Suppl. Figure 4j-p).

Transmission electron microscopy (TEM) analysis of control treated nerves at 5 dpi showed a low number of denervated myelin sheaths, identified as intact appearing myelin sheath without axonal content, in which the myelin degradation has not started yet (example in Fig. 3b, marked by black arrowheads in Fig. 3d, quantified in 3o). In contrast, a substantial amount of myelin debris was detected (example in Fig. 3b, marked by asterisks in Fig. 3d, quantified in 3o), indicating an advanced stage of myelin debris degradation. In contrast, mice receiving the acute PIO treatment showed delayed debris processing, specified by significant higher amounts of denervated myelin and lower numbers of myelin debris (Fig. 3h, o). In addition to this impairment in debris clearance, PIO treated mice showed a marked delay in early remyelination at 10 dpi (Fig. 3e, i, p), which persisted into the late remyelination phase at 21 dpi (Fig. 3f, j, p). Although the percentage of remyelinated fibers in PIO-treated animals had partially recovered by 21 dpi, G-ratio analysis revealed that the myelin sheaths remained thinner, as indicated by the increased G-ratio (Fig. 3q, r).

Despite the analysis of myelin morphology and remyelination, we assessed axonal regeneration over time in mice receiving acute PIO treatment. At 10 dpi, small caliber axons (mainly up to 2 μm of diameter, marked by red arrowheads in Fig. 3e) regrew within the injured nerve of control treated animals and gradually gained in diameter until 21 dpi (Fig. 3s). Interestingly, mice receiving the acute PIO treatment had a shift of axon diameter towards thinner axons (below 1 μm diameter, marked by orange arrowheads in Fig. 3i) at 10 and 21 dpi, indicating a delayed axonal regrowth after nerve injury (Fig. 3s), which might also account for the delayed remyelination and reduced myelin thickness (Fig. 3p-r). In line with this observation, we also found reduced expression of the pro-regenerative marker GAP-43 in the axons of mice with acute PIO treatment compared to control treated mice at 5 dpi (Suppl. Figure 5).

Next, we assessed whether the late PIO treatment (starting at 5 dpi) would have beneficial effects on regeneration and remyelination as indicated by our previous data. Here, the earliest analyzed timepoint was 14 dpi, since the treatment only started at 5 dpi and took place every second day. As hypothesized, we observed accelerated remyelination, with enhanced myelination at 14 dpi, which caught up at 21 dpi (Fig. 3k-n, t, u). Nevertheless, G-ratios of the myelinated fibers were significantly decreased at 21 dpi, indicating thicker myelin sheaths (Fig. 3v, w). Accordingly, we saw a slightly but significantly increased axonal diameter in mice receiving late PIO treatment at 14 dpi, which indicates accelerated axonal growth and might contribute to the increased remyelination (Fig. 3x).

In sum, we show that in vivo administration of PIO in mice with a sciatic nerve crush can have opposing effects on regeneration, depending on the time of treatment. While acute treatment after injury impaired axonal regeneration and remyelination, delayed treatment resulted in accelerated nerve regeneration.

### PIO treatment alters neuromuscular synapse reinnervation and motor functional recovery after nerve injury

**Fig. 4:**
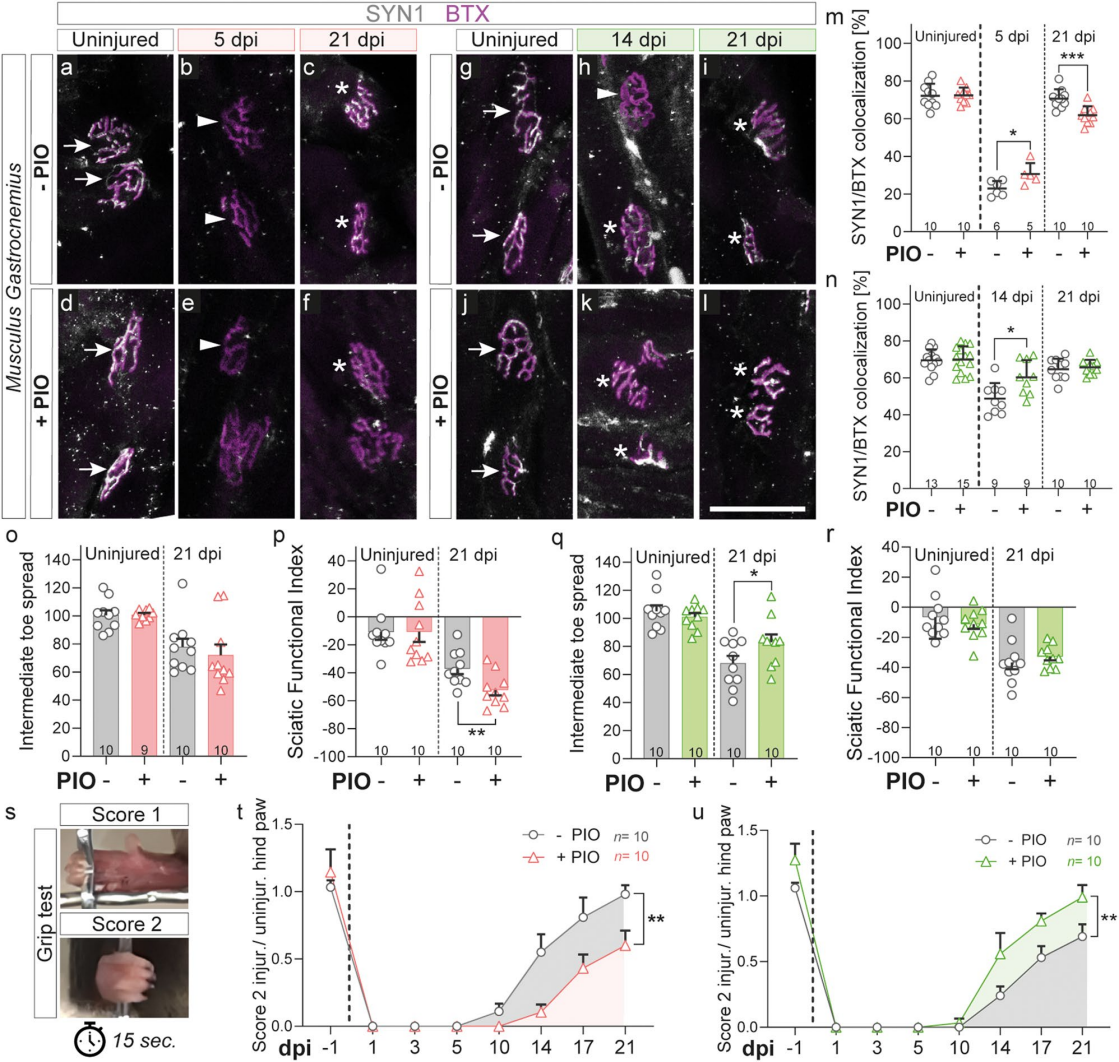
PIO treatment alters neuromuscular synapse reinnervation and motor functional recovery after nerve injury. **a**-**l**: Histology of neuromuscular junctions in gastrocnemius muscle sections stained with bungarotoxin (BTX, magenta) and Synaptophysin 1 (SYN1, white) at 5, 14, and 21 days post injury (dpi) and in uninjured control muscles. Arrows indicate fully innervated neuromuscular synapses, arrowheads show non-innervated synapses and stars fully re-innervated synapses. Mice received either “acute” PIO treatment (days 0–5 post injury daily; pictures **a**-**f**, red graphs) or “late” PIO treatment (days 5–21 post injury every second day; pictures **g**-**l**, green graphs). **m**: Quantification of SYN1/BTX colocalization at 5 and 21 dpi and in uninjured control muscles (pooled across timepoints) for “acute“ treatment. n: Quantification of SYN1/BTX colocalization at 14 and 21 dpi and in uninjured control muscles (pooled across timepoints) for “late“ treatment. **o**-**u**: Analysis of motor functional recovery and grip performance after nerve injury. **o**: Intermediate toe spread of uninjured and injured paws at 21 dpi for “acute” treatment. **p**: Sciatic functional index of uninjured and injured paws at 21 dpi for “acute” treatment. **q**: Intermediate toe spread of uninjured and injured paws at 21 dpi for “late” treatment. **r**: Sciatic functional index of uninjured and injured paws at 21 dpi for “late” treatment. **s**: Illustration of hind paw grip scores (“Score 1” and “Score 2”) used for analysis in the grip test. **t**: Grip test score 2 for injured paws normalized to uninjured paws over the regeneration time of 21 days in mice with “acute” treatment. **u**: Grip test score 2 for injured paws normalized to uninjured paws over the regeneration time of 21 days in mice with “late” PIO treatment. Each dot in the graphs in (**m**-**r**) represents a single mouse. *n* = 5–10 as indicated in the figures. Each dot in the graphs in (**t**-**u**) represents the mean of 10 mice per timepoint. Statistical significance was determined using a two-sided Mann-Whitney test. Error bars represent mean with SD (**m**, **n**) or mean with SEM (**o**-**r**, **t**, **u**). Statistical significance is indicated by asterisks (* P < 0.05, ** P < 0.005, *** P < 0.001). Scale bar in (**l**) is 50 µm (applies to (**a**-**l**)). BTX, Bungarotoxin; dpi, days post injury; PIO, Pioglitazone; SYN1, Synaptophysin 1

Above, we described beneficial or detrimental effects of PIO treatment on nerve regeneration depending on the temporal treatment paradigm. As a next step, we investigated whether those changes in nerve regeneration were also reflected in altered target muscle reinnervation (Fig. 4). Therefore, we assessed the degradation and reinnervation of neuromuscular junctions (NMJs) in the gastrocnemius muscle of mice with a sciatic nerve crush, receiving either acute or late PIO treatment. Here, NMJs were histologically detected by bungarotoxin (BTX) labeling of the post-synapse on the muscle and synaptophysin (SYN1) labeling of the pre-synaptic innervation.

Sciatic nerve injury resulted in denervation of NMJs of the gastrocnemius muscle indicated by depletion of the SYN1 signals at 5 dpi, while the post-synaptic BTX^+^ part of the NMJ is preserved (Fig. 4b, m). In line with our previous findings of delayed debris clearance, SYN1 signals were not as efficiently depleted in mice receiving acute PIO treatment at 5 dpi (Fig. 4e, m). Further, reinnervation of the NMJs at 21 dpi was delayed in PIO treated mice (Fig. 4c, f, m), which is in accordance with the impaired nerve regeneration described above. Interestingly, late PIO treatment starting at 5 dpi, accelerated NMJ reinnervation after sciatic nerve crush, with reinnervation almost reaching pre-injury levels already at 14 dpi as opposed to control treated mice reaching this level only at 21 dpi (Fig. 4g-l, n).

Since muscle reinnervation in mice was altered depending on the timing of PIO treatment, we went on to assess functional motor regeneration. In doing so, we used CatWalk XT analysis to evaluate changes in gait parameters during voluntary walking over the time course of regeneration. Here, the placement of the injured paw could only be evaluated at 21 dpi, since at all earlier time points paw pressure was too weak to be detected by the CatWalk XT system. Nevertheless, analysis at 21 dpi showed a slightly impaired motor regeneration especially in the sciatic functional index (SFI) in mice that received an acute PIO treatment (Fig. 4o, p). Mice receiving the late PIO treatment on the other hand, showed slightly improved motor regeneration, especially regarding the intermediate toe spread of the affected paw (Fig. 4q, r).

Finally, we wanted to assess a motor task more demanding than merely voluntary walking. To do so, we employed a so-called grip test, where mice were placed on a grid, the grid was then slowly inverted and mice were filmed for one minute from the top. Then, the ability of the affected paw to grab the rods of the grid was scored by 0 (paw cannot grab at all), 1 (paw can only grab using the toes) or 2 (the whole paw is used for grabbing) during a period of 15 s of constant movement (Fig. 4s). Here, mice treated acutely with PIO showed a clear reduction in rod-grasping efficiency compared to controls (Fig. 4t), in parallel with the impaired axonal regeneration observed at the molecular level. In contrast, late PIO treatment improved motor regeneration significantly compared to control treated mice (Fig. 4u), which again is in line with the improvement in nerve regeneration observed on histological level.

Taken together, assessment of motor recovery in mice receiving PIO after nerve injury showed that acute PIO treatment resulted in impaired muscle reinnervation and motor functional recovery. As opposed to these findings, late PIO treatment enhanced both muscle reinnervation and motor functional recovery. Those observations were in accordance with the hampered or improved nerve regeneration in dependence to the timing of PIO treatment described above on a molecular/histological level (Fig. 3).

### PIO treatment alters epidermal reinnervation and sensory functional recovery after nerve injury

**Fig. 5:**
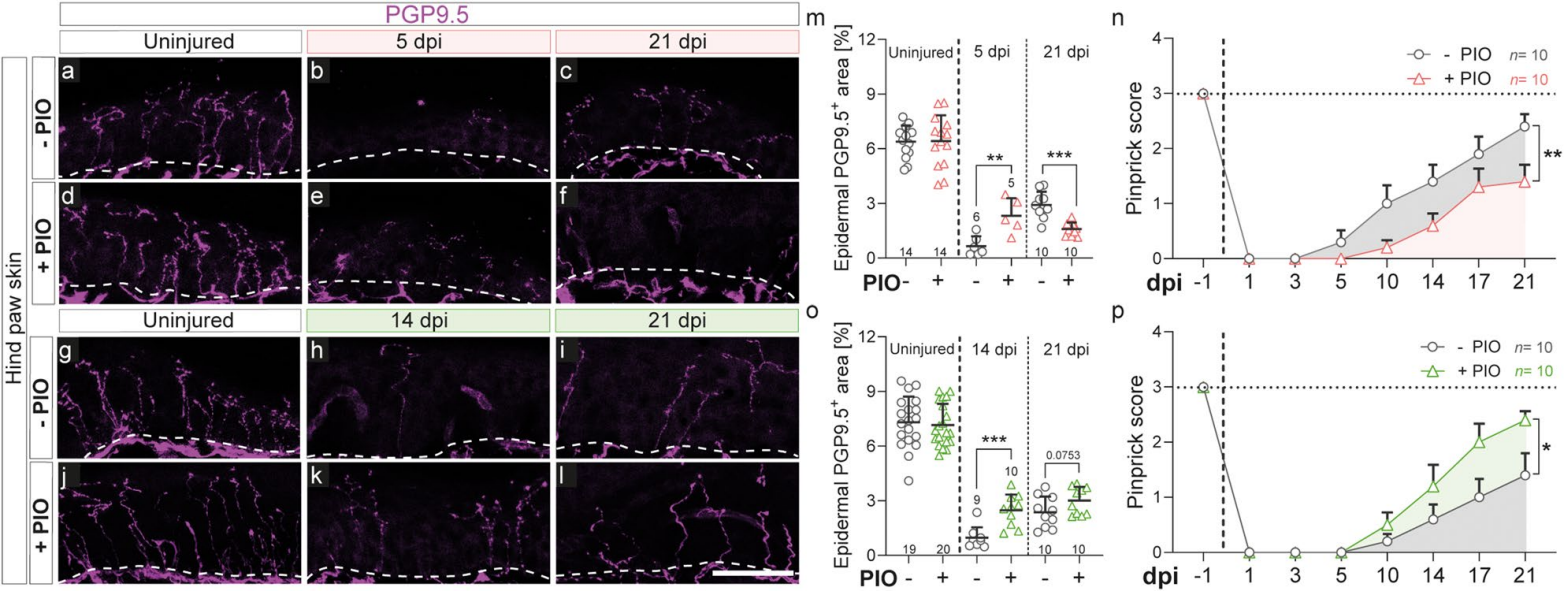
PIO treatment alters epidermal reinnervation and sensory functional recovery after nerve injury. **a**-**l**: Histological staining of PGP9.5 showing epidermal innervation in the hind paw interdigital skin in uninjured paws or at 5, 14, and 21 days post injury. Dashed lines indicate the boundary between the epidermis and dermis. Mice received either “acute” PIO treatment (days 0–5 post injury daily; pictures **a**-**f**, red graphs) or “late” PIO treatment (days 5–21 post injury every second day; pictures **g**-**l**, green graphs). **m**: Quantification of intra-epidermal PGP9.5^+^ area at 5 and 21 dpi and in uninjured control paws (pooled across timepoints) for “acute” treatment. **n**: Pinprick scores over 21 days post injury in mice with “acute” treatment. **o**: Quantification of intra-epidermal PGP9.5^+^ area 14, and 21 dpi and in uninjured control paws (pooled across timepoints) for “late” treatment. **p**: Pinprick scores over 21 days post injury in mice with “late” treatment. Each dot in the graphs in (**m**) and (**o**) represents a single mouse. *n* = 5–10 as indicated in the figures. Each dot in (**n**) and (**p**) represents the mean of 10 mice per timepoint. Statistical significance was determined using a two-sided Mann-Whitney test. Error bars represent mean with SD (**m**, **o**) or mean with SEM (**n**, **p**). Statistical significance is indicated by asterisks (* *P* < 0.05, ** *P* < 0.005, *** *P* < 0.001). Scale bar in (**l**) is 50 μm (applies to (**a**-**l**)). dpi, days post injury; PIO, Pioglitazone

After analyzing motor recovery, we went on to evaluate sensory recovery of the hind paw as a target tissue of the sciatic nerve. Therefore, we collected skin from the middle interdigital part of the control and injured hind paws and performed histological analysis using PGP9.5 as marker for nerve endings within the epidermis (Fig. 5). At 5 dpi, PGP9.5 was almost absent in control treated mice, indicating that nerve endings are fully degenerated due to the sciatic nerve injury (Fig. 5a-b, m). In contrast, fragments of PGP9.5 staining were still clearly visible in mice which received the acute PIO treatment at 5 dpi (Fig. 5e, m), in line with the previously observed delay in nerve debris removal (Fig. 3h, o). When epidermal reinnervation was evaluated at 21 dpi, control treated animals indeed showed a certain degree of regeneration, while mice acutely treated with PIO had barely any PGP9.5 signal detectable at this time point (Fig. 5c, f, m). In contrast, late PIO treatment led to complete epidermal reinnervation already by 14 dpi, whereas control-treated animals required 21 days to nearly achieve the same level of reinnervation (Fig. 5g-l, o).

Next, we wanted to investigate, if the above-described alterations in epidermal reinnervation also reflect on changes in sensory functional recovery. Therefore, we performed a pinprick test at the same central interdigital paw position as used for the histological analysis and evaluated the response of the mice to this pain stimulation. As expected on the basis of the histological results, mice acutely treated with PIO displayed significantly reduced sensitivity to stimulation compared to control treated mice (Fig. 5n). In contrast, late PIO treatment resulted in improved sensing of painful stimuli (Fig. 5p), which was also in line with the histological findings.

All in all, acute PIO treatment impaired, while late PIO treatment accelerated epidermal reinnervation and sensory functional recovery after sciatic nerve crush.

### PIO treatment increases mitochondrial abundance in axons and SCs in vivo and ex vivo

So far, our results demonstrated that acute PIO treatment has detrimental effects on nerve regeneration, while late PIO treatment improved axonal regeneration, target tissue reinnervation, remyelination and functional recovery of injured nerves. Due to the above-described PIO-mediated induction of lipid metabolism and suppression of SC repair phenotype, we had anticipated that late PIO treatment might improve remyelination. Nonetheless, this alone might not fully account for the generally improved axonal regeneration.

To uncover a potential molecular mechanism underlying the altered axonal outgrowth observed in PIO-treated mice across the two different treatment regimes, we analyzed the expression of regeneration-associated genes (RAGs) in the dorsal root ganglia (DRG) of mice that received systemic PIO administration. In agreement with previous results [39], injury-induced upregulation of RAG expression (e.g. *Atf3*,* cJun*,* Sox11*,* Timp1*,* Gap43*) was observed in the DRGs of injured nerves (Suppl. Figure 6). PIO treatment did not significantly alter this expression irrespective of the time of administration, suggesting that neuronal RAG responses were not majorly affected by systemic PIO application. Furthermore, PIO treatment of cultured primary DRG neurons did not significantly alter neurite outgrowth (Suppl. Figure 7), which indicates that improved axonal outgrowth in mice receiving late PIO treatment rather depends on a non-cell-autonomous mechanism.

**Fig. 6:**
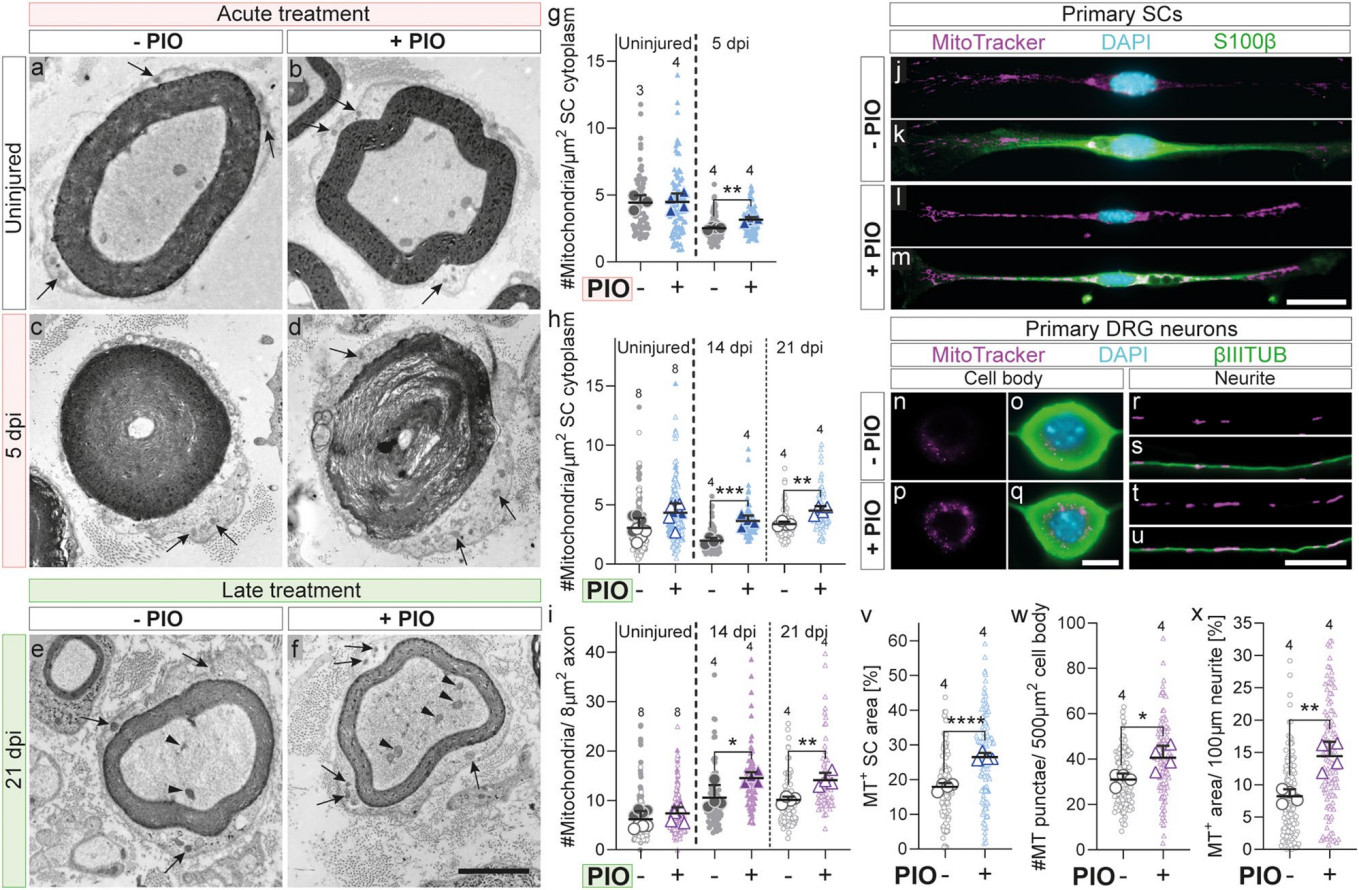
PIO treatment increases mitochondrial abundance in axons and SCs in vivo and in vitro. **a**-**f**: TEM images of murine sciatic nerve cross-sections in uninjured control samples and at 5 and 21 days post injury. Mice received either “acute” PIO treatment (+ PIO, days 0–5 post injury daily; pictures a-d; quantification **g**) or “late” PIO treatment (+ PIO, days 5–21 post injury every second day; pictures **e**, **f**; quantifications **h**,**i**), or corresponding vehicle treatment with DMSO (- PIO). Arrows indicate mitochondria within SCs, arrowheads indicate mitochondria within axons. **g**: Quantification of mitochondrial numbers per SC cytoplasm in murine uninjured sciatic nerves and at 5 dpi for “acute” treatment. **h**, **i**: Quantification of mitochondrial numbers per SC cytoplasm (**h**) and per axon (**i**) in murine uninjured sciatic nerves (pooled across timepoints) and 21 dpi for “late” treatment. **j**-**m**: Primary murine SCs treated with PIO (+ PIO) or DMSO (- PIO) for 5 consecutive days stained for SC marker (S100β) and mitochondria (MitoTracker (MT)). **n**-**u**: Primary DRG neurons treated with PIO (+ PIO) or DMSO (- PIO) for 5 consecutive days, stained for neuronal marker (βIIITUB) and mitochondria (MitoTracker) in neuronal cell bodies (**n**-**q**) and in neurites (**r**-**u**). **v**: Quantification of MitoTracker^+^ area per SC. **w**: Quantification of Mitotracker^+^ fluorescence punctae per DRG cell body. **x**: Quantification of MitoTracker^+^ area per neurite. Each large dot in (**g**-**i**) represents the mean value of a single mouse, while small background dots indicate individual measurements; *n* = 3–4. Each large dot in (**v**-**x**) represents the mean value of one biological replicate, and small background dots indicate individual measurements; *n* = 4 biological replicates with SCs or DRGs isolated from a single mouse. All error bars represent mean with SD. Statistical significance was determined using a two-sided T-test. Statistical significance is indicated by asterisks (* *P* < 0.05, ** *P* < 0.005, *** *P* < 0.001). Scale bar in (**f**) is 2 μm (applies to **a**-**f**). Scale bar in (**m**) is 20 μm (applies for **j**-**m**). Scale bars in (**q**) and (**u**) are 10 μm (apply to **n**-**u**). dpi, days post injury; DRG, dorsal root ganglion; MT, MitoTracker; PIO, Pioglitazone; SC, Schwann cell; TEM, transmission electron microscopy

The analysis of the commonly upregulated GO terms in PIO treated nerve explants SCs, had revealed that PIO activated GO terms related to mitochondria (Suppl. Figure 2b). PPARγ is a known regulator of mitochondrial function and energy metabolism and has been previously proposed as therapeutic target for the improvement of energy metabolism in neurological disorders [40]. In addition, nerve regeneration was previously reported to have high energy demands and require drastic metabolic adaptations [13]. Hence, as a next step, we decided to analyze mitochondrial abundance in the nerves of mice receiving PIO treatment. In doing so, we found that PIO treated mice had elevated mitochondrial content in the SCs of injured nerves at 5 dpi in the acute treatment and at 14 and 21 dpi in the late treatment (Fig. 6a-h). Notably, in mice that had received the late treatment, mitochondria counts were also increased in the regenerating axons of injured nerves at 14 and 21 dpi (Fig. 6e-f, i).

Next, we wanted to analyze whether this elevated mitochondrial content is regulated by a cell autonomous process, or by a paracrine effect due to the systemic PIO administration. We therefore took primary murine SCs or DRG neurons in culture and treated them with PIO for 5 days, prior to analyzing mitochondrial abundance. Interestingly, PIO treatment enhanced mitochondrial abundance in the SC bodies and processes, as well as DRG neuron somata and neurites (Fig. 6j-x). This shows that PIO treatment in mice can have a direct effect on SCs and neurons, thereby elevating mitochondria content. In order to investigate if this process is conserved among species, we isolated primary human SCs, treated them with PIO and analyzed the responses. Gene expression analysis confirmed PIO-mediated activation of *PPARγ* and *mPPARγ* in human SCs, as well as increase in mitochondrial content (Suppl. Figure 7), underlining the potential of PPARγ regulation as a therapeutic target in peripheral nerve injuries.

Taken together, our data show that PIO treatment does not seem to influence axonal regeneration by altering RAG expression in affected DRG neurons. Instead, we discovered an elevated abundance of mitochondria in SCs and axons of regenerating nerves in mice receiving late PIO treatment.

### PIO treatment enhances mitochondrial respiration, glycolysis and lipid metabolism in SCs

**Fig. 7:**
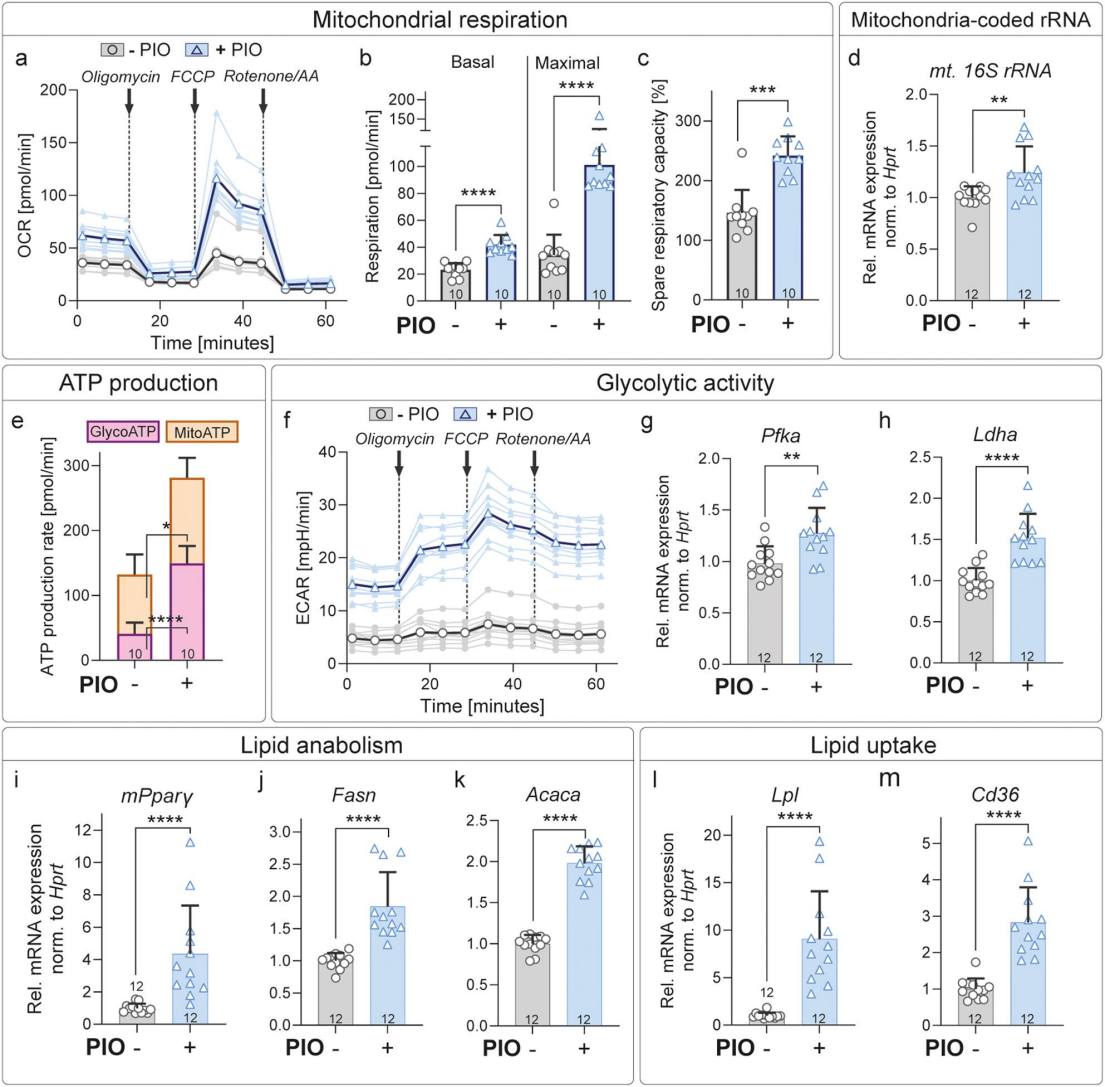
PIO treatment enhances mitochondrial respiration, glycolysis, as well as lipid synthesis and uptake in SCs. **a**-**c**, **e**-**f**: Seahorse XT respirometry in primary murine SCs treated with either PIO (+ PIO, blue) or DMSO (- PIO, gray) for 5 consecutive days. **a**: Measurement of oxygen consumption rate (OCR) for individual biological replicates treated with PIO (+ PIO; light blue lines) or DMSO (- PIO; light gray lines), with mean values for each treatment group (dark blue and dark gray, respectively). **b**: Quantification of basal and maximal respiration. **c**: Quantification of spare respiratory capacity. **d**: qPCR analysis of mitochondria-coded 16 S rRNA in primary murine SCs treated with either PIO (+ PIO, blue) or DMSO (- PIO, gray) for 5 consecutive days. **e**: Quantification of ATP production derived from glycolysis (GlycoATP, pink) and mitochondrial respiration (MitoATP, orange). **f**: Measurement of extracellular acidification rate (ECAR) for individual biological replicates treated with PIO (+ PIO; light blue lines) or DMSO (- PIO; light gray lines), with mean values for each treatment group (dark blue and dark gray, respectively). **g**-**m**: qPCR analyses of genes related to glycolysis (**g**-**h**), lipid anabolism (**i**-**k**), and lipid uptake (**l**, **m**) in primary murine SCs treated with either PIO (+ PIO, blue) or DMSO (- PIO, gray) for 5 consecutive days. For all qPCR analyses, gene expression in DMSO-treated SCs was normalized to one and fold changes were calculated for PIO-treated samples. Biological replicates analyzed: *n* = 10 in (**a**-**c**, **e**-**f**) and *n* = 12 in (**d**, **g**-**m**). Each dot represents primary SCs isolated from a single mouse. Statistical significance was determined and two-sided Mann-Whitney test. All error bars show mean with SD. Statistical significance is indicated by asterisks (* *P* < 0.05, ** *P* < 0.005, *** *P* < 0.001, **** *P* < 0.0001). AA: Antimycin A; ATP, Adenosine triphosphate; ECAR, Extracellular acidification rate; FCCP, carbonyl cyanide-*p*-trifluoromethoxyphenylhydrazone; Glyco, glycolytic; Mt., mitochondrial; OCR, oxygen consumption rate; PIO, Pioglitazone; SC, Schwann cell

To determine whether the increase in mitochondrial content upon PIO treatment (Fig. 6) was accompanied by metabolic adaptations, we further investigated the metabolic profile of primary murine SCs. Therefore, we used primary SC cultures, treated them with PIO for 5 days and performed Seahorse XF measurements to investigate cellular energy metabolism. As expected, a significantly enhanced oxygen consumption rate (OCR) in SCs treated with PIO was found compared to control treated SCs (Fig. 7a). Furthermore, basal and maximal respiration as well as spare respiratory capacity were also elevated by PIO treatment, indicating enhanced respiratory capacity (Fig. 7b-c). Apart from enhanced mitochondria respiration we found slightly but significantly increased expression of the mitochondria-coded 16 S rRNA in PIO treated SCs (Fig. 7d), pointing at enhanced mitochondrial counts. This was in line with our previous finding in SC cultures (Fig. 6j-m, v).

Subsequently, we went on to analyze potential effects of PIO treatment on the glycolytic function of SCs. Our Seahorse XF measurements indicated that in untreated cultured SCs, mitochondrial ATP production represents the major energy source, although glycolytic ATP still contributes a considerable portion of total ATP (Fig. 7e). Upon PIO treatment, glycolytic activity – as reflected by the extracellular acidification rate (ECAR) – and glycolytic ATP production were markedly increased (Fig. 7e-f). Indeed, gene expression of the enzyme *phosphofructokinase I* (*Pfka)* – the key regulating enzyme of glycolytic activity – was elevated in PIO treated SCs (Fig. 7g). In addition, *lactate dehydrogenase A* (*Ldha)* gene expression was induced by PIO, indicating not only enhanced glycolytic activity but also enhanced turnover of pyruvate into lactate in SCs treated with PIO (Fig. 7h).

Finally, we analyzed gene expression related to lipid anabolism and uptake. As previously shown, lipids have a crucial contribution to the overall metabolism of SCs [41], and PPARγ is a major regulator of this pathway. Hence, we aimed to investigate to what extent adaptations on mitochondrial respiration are also accompanied by changes of lipid metabolism. Gene expression analysis revealed elevated expression of genes involved in lipid anabolism (e.g. *mPparγ*,* Fasn*,* Acaca;* Fig. 7i-k) and uptake (e.g. *Lpl*,* Cd36;* Fig. 7l-m) indicating an increase in lipid synthesis in PIO treated SCs.

These experiments demonstrate that PIO treatment enhances energy homeostasis in SCs by promoting mitochondrial respiration, stimulating glycolytic activity, and in turn, extracellular acidification, and increasing mRNA expression of lipid-importing and synthetizing enzymes. These metabolic adaptations may contribute to improved axonal regeneration and subsequent remyelination.

**Fig. 8:**
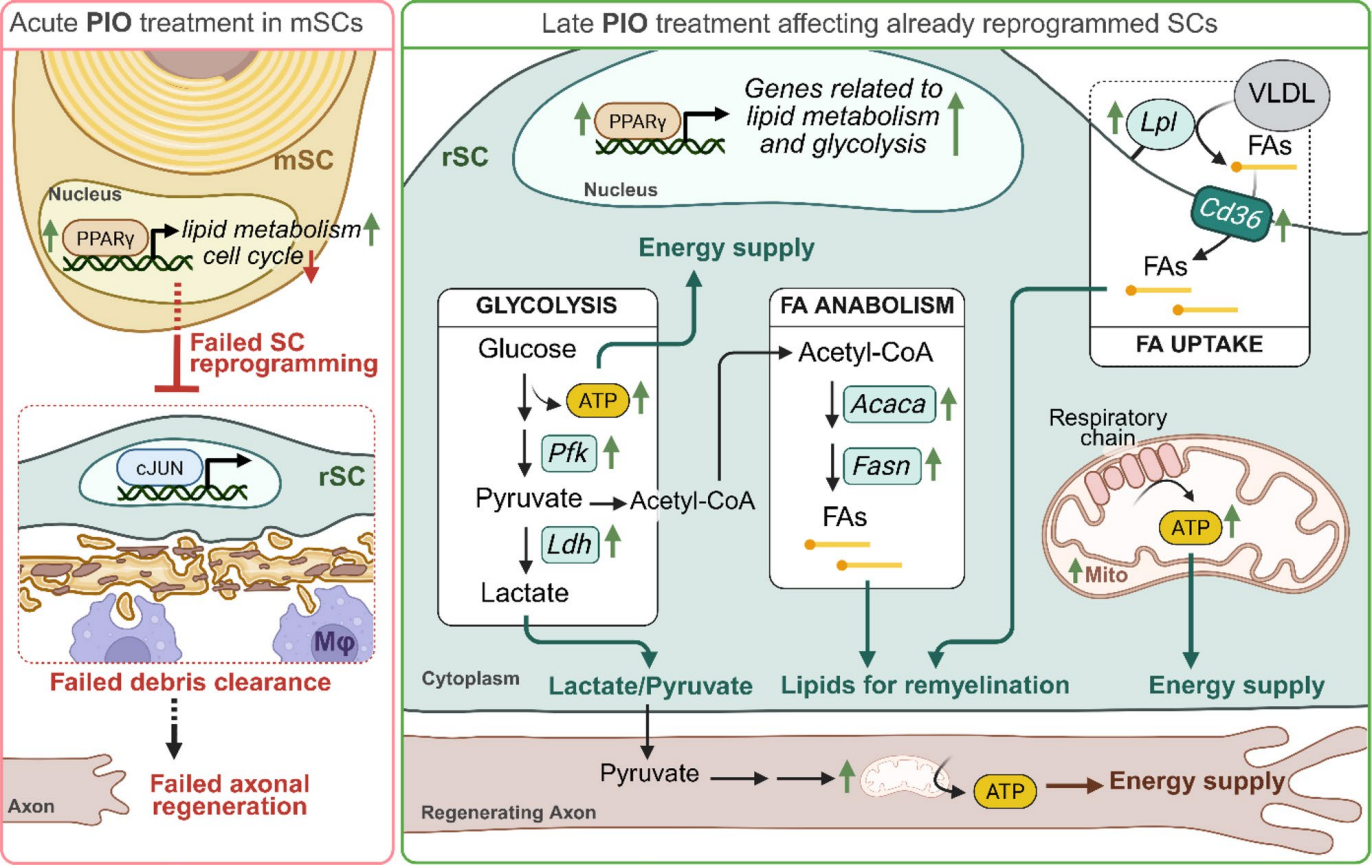
Proposed mechanism of acute and late PIO treatment on regeneration of injured nerves. Acute PIO treatment (red box) induces lipid metabolism gene expression and downregulates cell cycle-associated genes in SCs, impairing their transition into the repair phenotype. As a result, the inflammatory response is blocked. This leads to delayed axonal and myelin clearance, hindering the overall nerve regeneration process. In contrast, late PIO treatment (green box) in already dedifferentiated repair SCs enhances PPARγ activity and thereby glycolysis, lipid anabolism and uptake. It also increases mitochondrial abundance and ATP production in SCs while boosting mitochondrial abundance in regenerating axons. These metabolic adaptations seem to promote axonal outgrowth, remyelination, and overall functional recovery in mice. These temporal effects underscore the dual role of lipid metabolism adaptations modulating metabolic reprogramming and SC plasticity during nerve repair. ATP, Adenosine triphosphate; FA, fatty acid; mSC, myelinating Schwann cell; PIO, Pioglitazone; rSC, repair Schwann cell; SC, Schwann cell. Created with BioRender.com

Overall, this study reveals a time-dependent effect of Pioglitazone on nerve regeneration: acute treatment disrupts the physiological injury response (summarized in Fig. 8, left side), whereas late treatment induces metabolic adaptations that ultimately enhance nerve recovery (summarized in Fig. 8, right side).

## Discussion

In this study, we investigated PPARɣ as a molecular target for modulating metabolic functions in injured nerves. Using a sciatic nerve crush model in mice, we applied PIO treatment either acutely (days 0–5, daily) or late (days 5–21, every other day) to address the distinct metabolic demands of Wallerian degeneration and subsequent regeneration. By comparing molecular and functional outcomes, we aimed to identify the critical window and mechanism through which PPARɣ activation influences nerve repair.

First, we investigated SC responses to PIO treatment ex vivo using an unbiased RNAseq approach (Fig. 1, Suppl. Figure 1–2). This confirmed that PIO treatment results in a strong upregulation of PPAR signaling and lipid metabolism-related pathways – a response previously observed by us in human and murine SCs [12] and widely described in other cell types [42]. Our ex vivo findings were also corroborated in vivo by the increased abundance of PPARγ and enhanced accumulation of lipid droplets in injured nerves of PIO treated mice, as compared to control treated mice (Fig. 2a-h, q-r). This lipid droplet accumulation may result from a lipid overload within the SCs due to the combination of halted myelination [43] and boosted lipid metabolism in PIO treated mice. Further, lipid droplet accumulation might also indicate that SCs struggle to efficiently process excess lipids taken up by myelinophagy. Although speculative at the moment, this interpretation is supported by our observation that mice subjected to acute PIO treatment exhibited delayed clearance of myelin debris (Fig. 3d, h, o).

Besides regulating lipid metabolism, previous data suggest that PIO mediated PPARɣ activation mitigates inflammation by suppressing NF-κB signaling [44] and thereby the release of inflammatory mediators in disease models such as traumatic brain injury [45]. However, our own results present an interesting paradox: while PIO treatment mainly induced inflammation associated gene expression in our ex vivo nerve injury model, it drastically reduced immune cell infiltration in vivo (Figs. 1a-b and 2i-l and s). This discrepancy may be attributed to the absence of peripheral immune cells in the ex vivo nerve explant system (meaning that measured responses mostly come from SCs and other resident cell types within the explant), whereas in vivo, systemically applied PIO is likely to additionally exert direct immunomodulatory effects on peripheral immune cells.

Among the KEGG pathways with the strongest downregulation after PIO treatment in vitro/ex vivo, RNAseq analysis revealed altered cell cycle-associated processes, as well as the Hippo and Wnt signaling pathways (Fig. 1c, f). These pathways have been previously implicated in SC functions, including proliferation, myelination, and injury response [46–48]. Thus, their modulation may affect nerve regeneration in vivo, making them promising targets for future research.

One further SC function, which we recently demonstrated to be affected by PIO treatment in an ex vivo system is dedifferentiation of SCs into the repair phenotype after injury [12]. Consistently, systemic PIO administration in vivo led to a similar effect, reducing the expression of the prototypical repair SC marker cJUN (Fig. 2m-p, t). Notably, we previously demonstrated that human nerve explants respond to pharmacological PPARɣ modulation by PIO, with effects comparable to those in mice regarding SC reprogramming and the above-described lipid gene regulation [12]. This underscores the conserved role of PPARɣ in SCs and highlights the translational relevance of PIO treatment in humans.

Taking all these findings into account, we hypothesized that PIO treatment immediately after injury (here termed acute PIO treatment) will be detrimental to nerve regeneration, by blocking the development of repair SCs, the clearance of nerve debris and as a consequence overall nerve regeneration (see also Fig. 8 left side). Indeed, our in *vivo* data from mice being treated with PIO immediately after injury show delayed debris removal (Fig. 3d, h, o), impaired axonal regeneration and remyelination (Fig. 3e-f, i-j, p-s), reduced reinnervation of target tissues (i.e. muscle reinnervation in Fig. 4a-f, m and hind paw skin reinnervation in Fig. 5a-f, m) as well as delayed motor and sensory functional recovery (Figs. 4o-p and t and 5m-n). Our findings demonstrate how perturbation of the initial process of Wallerian degeneration can affect the overall long-term nerve regeneration, which is in accordance with previous studies [49].

On the other hand, the RNAseq results indicated upregulation of lipid metabolism in PIO-treated SCs and nerve explants, which might promote the myelinating over the repair SC phenotype. Based on this observation, we speculated that PIO treatment might be beneficial for nerve regeneration if applied in a phase when SCs are already reprogrammed into repair SCs. To evaluate this, we administrated PIO as a “late” treatment (from day 5 onward). Consistent with our hypothesis, late PIO treatment resulted in accelerated target tissue reinnervation and axonal remyelination (Fig. 3k-n, u), and furthermore improved sensory and motor recovery on functional level (Figs. 4q and u and 5p). Interestingly, despite the fact that control-treated mice seemed to catch up on molecular level at 21 dpi, functional recovery of PIO treated mice was still far better at this time point. This could indicate better signal propagation due to the increased myelin thickness or – although still speculative – mitigated muscle atrophy due to accelerated regeneration. Of note, overall functional recovery in mice subjected to late PIO treatment was poorer compared to those with acute treatment. This may be a result of the prolonged treatment period (over 17 days as compared to 5 days in acute treatment) in mice receiving the late PIO treatment and thereby increased handling-related stress.

Interestingly, late PIO treatment did not just positively influence remyelination, but also axonal regeneration and target tissue reinnervation (Figs. 3x, 4g-l and n and 5g-l and o). Those effects probably cannot just be attributed to the enhanced shift of repair SCs toward the myelinating phenotype. Hence, we tried to identify further PIO-dependent mechanisms that might be involved in nerve recovery. Since PIO treatment was administered systemically, it is likely that cell types beyond SCs also contributed to the observed changes in regeneration. First of all, we analyzed potential effects of PIO directly on the expression of RAGs in DRG neurons, which might explain altered axonal outgrowth [39]. However, our data revealed that PIO treatment did not significantly alter RAG gene expression in DRG neurons following injury (Suppl. Figure 6), which was in line with the unaltered neurite outgrowth of cultured, PIO-treated DRG neurons (Suppl. Figure 7).

RNAseq analysis of commonly upregulated pathways in PIO-treated nerve explants and SCS had revealed that one of the most robust SC responses to PIO-treatment were related to mitochondrial functions (Suppl. Figure 2b). PPARγ is a known modulator of mitochondrial function and energy homeostasis [50] and its agonist PIO has been proposed to enhance mitochondrial biogenesis and improve energy metabolism in neurological disorders [40, 51, 52]. Hence, we decided to analyze mitochondrial abundance within the injured nerve next. It was previously demonstrated that mitochondrial abundance in axons decreases during the initial degeneration phase but subsequently increases during the regeneration process in order to promote recovery [53]. Thus, we conjectured that PIO treatment of injured nerves during the regeneration phase could potentially improve metabolic homeostasis. Indeed, our data revealed that late PIO treatment increased mitochondrial content in both axons and SCs (Fig. 6e-f, h-i). This dual impact of PIO on both axons and SCs was further validated through in vitro treatment of primary murine SCs or DRG neurons (Fig. 6j-x), indicating that PIO treatment most likely directly influences SC and neuronal function. Importantly, primary human SCs also responded to PIO treatment with increased mitochondrial abundance (Suppl. Figure 8), once again underlining the therapeutic potential of PPARγ modulation in humans. Enhanced mitochondrial content in axons could be an indicator of increased ATP supply, which might contribute to the enhanced regeneration. Nevertheless, our in vitro results in cultured DRG neurons show, that enhanced mitochondrial content alone is probably not sufficient to increase neurite outgrowth (Suppl. Figure 7). Despite the potential beneficial effects of elevated axonal ATP, enhancing ATP supply in SCs might further contribute to the accelerated remyelination after nerve injury. Of note, also the acute PIO treatment in mice led to enhanced mitochondrial content in SCs of injured nerves (Fig. 6a-d, g). This is in line with the RNAseq data, showing upregulated mitochondria-related GO terms in PIO-treated primary SCs (resembling the late phase treatment) and in nerve explants (resembling the acute phase treatment; Suppl. Figure 2b).

Since SCs are energetically coupled to axons to provide support [54], we sought to analyze metabolic adaptations in PIO-treated SCs in more detail. Seahorse XF analysis confirmed that PIO increased mitochondrial respiration and ATP production (Fig. 7a-c, e). Notably, PIO treatment not only increased mitochondrial ATP production, but also a strikingly elevated glycolysis-derived ATP supply (Fig. 7e-f) further improving energy homeostasis in SC of regenerating nerves. Our findings are further supported by literature, where PIO was described to enhance glycolysis and mitochondrial ATP production in a model of cardiac dysfunction [55]. In accordance with the elevated glycolytic ATP production, expression of key regulating enzymes in glycolysis (e.g. *Pfka*) and lactate production (e.g. *Ldha)* were induced (Fig. 7g, h). Although speculative at the moment, increased glycolysis and LDH (lactate dehydrogenase) abundance might indicate higher abundance of the metabolites pyruvate and lactate in PIO treated SCs. These metabolites could be transported to the regenerating axons, providing additional metabolic support – besides the increased mitochondrial abundance – for the regeneration process, as previously described [54]. Hence, PIO-mediated metabolic adaptations in SCs might further contribute to improved axonal regrowth, in addition to the enhanced mitochondrial content in axons themselves (see also Fig. 8 right side).

Given that metabolism, ATP production and remyelination seem to be closely associated to lipid metabolism in SCs [13, 41, 56, 57], we wanted to validate the above described adaptations in lipid metabolism (Fig. 1) also in this experimental setup and found upregulated genes for FA anabolism (*mPparγ*,* Fasn*,* Acaca*) and lipid uptake (*Lpl*,* Cd36*) (Fig. 7i-m). FA synthesis is known to positively influence remyelination in injured nerves [58, 59]. Further, SC lipoprotein lipase (LPL) was shown to produce FAs, which are then taken up into the SC through CD36, thereby contributing to FA supply for the remyelination [60]. These results signpost that PIO treatment on one hand enhances energy supply, on the other hand may improve FA abundance in SCs, resulting in accelerated remyelination.

Notably, mitochondrial dysfunction is closely linked to various neuropathies, characterized by neuropathic pain, axonal loss, and demyelination [61–63]. The above described PIO-mediated improvement of mitochondrial abundance and function in axons and SCs may therefore also offer therapeutic benefits for neuropathies. Indeed, preliminary clinical data in patients with painful diabetic neuropathy already suggest reduced pain and improved fiber regeneration under PIO treatment [62].

In summary, our findings reinforce the critical role of dynamic metabolic adaptation in nerve repair, as previously described [13], and identify PIO as a promising pharmacological candidate. Notably, our study accounts for the often-overlooked dual role of SCs after nerve injury: the early repair SC phenotype, which promotes nerve debris clearance, versus later SC functions that support axonal outgrowth and remyelination. Given the distinct requirements of these phases in regeneration, the timing of treatment administration is crucial. As demonstrated for PIO in this study, a pharmacological intervention may be beneficial within a specific therapeutic window but could be ineffective or even detrimental if administered outside this period.

### Limitations

Systemic drug administration, as applied in this study with pioglitazone, limits the ability to pinpoint the specific cellular contributors to the observed effects. To disentangle these mechanisms, cell type-specific PPARɣ knockout mouse models would offer valuable insights into the cell-autonomous functions of PPARɣ signaling. Nonetheless, our in vitro analyses indicate that the altered regenerative potential of nerves arises from the combined responses of multiple cell types – SCs, neurons, and inflammatory cells – rather than from a single cell population. Moreover, further studies are needed to elucidate how enhanced metabolic adaptations and increased mitochondrial content actually influence axonal outgrowth and regeneration. Finally, although we previously demonstrated that human Schwann cells respond to PIO treatment with changes in lipid metabolism, it remains unclear whether broader metabolic adaptations also occur in human SCs and whether these effects translate in vivo to modify nerve regeneration.

## Conclusion

Peripheral nerve injuries represent a major clinical challenge due to limited therapeutic options that effectively enhance regeneration. This study identifies the nuclear receptor PPARɣ as a time-sensitive regulator of Schwann cell function during nerve repair. Using the PPARɣ agonist pioglitazone, we demonstrate that early intervention disrupts the repair program of Schwann cells, while delayed administration improves metabolic homeostasis, remyelination and functional recovery. These findings highlight the importance of timing in metabolic adaptations during nerve regeneration and suggest that strategic activation of PPARɣ may offer a novel approach to improve outcomes after nerve injury.

## Supplementary Information

Below is the link to the electronic supplementary material.

Supplementary Material 1

## Data Availability

We intend to deposit our RNA-seq data to the GEO repository to make them available for the research community. Unfortunately, due to the current lapse in government funding, the upload cannot be processed at the moment. Nevertheless, the data are ready to be uploaded as soon as the GEO will allow it. Meanwhile, the tables including all processed data can be accessed as a supplement to this manuscript.

## Abbreviations

Dpi: Days post injury
DRG: Dorsal root ganglia
LD: Lipid droplet
PIO: Pioglitazone
PNI: Peripheral nerve injury
PNS: Peripheral nervous system
PPARɣ: Peroxisome proliferator-activated receptor gamma
SC: Schwann cell
SFI: Sciatic functional index
