# Supplementary material for: Pioglitazone modulates metabolic adaptation and peripheral nerve regeneration after injury
Source: Mol Neurodegener. 2025 Dec 1;20:123. doi: 10.1186/s13024-025-00913-1 (PMC12670764; doi:10.1186/s13024-025-00913-1)
Supplement: Supplementary file 1 — Supplementary Material 1 [file 13024_2025_913_MOESM1_ESM.docx]

**Supplementary Information**

Pioglitazone modulates metabolic adaptation and peripheral nerve regeneration after injury

Seitz *et al.* 2025


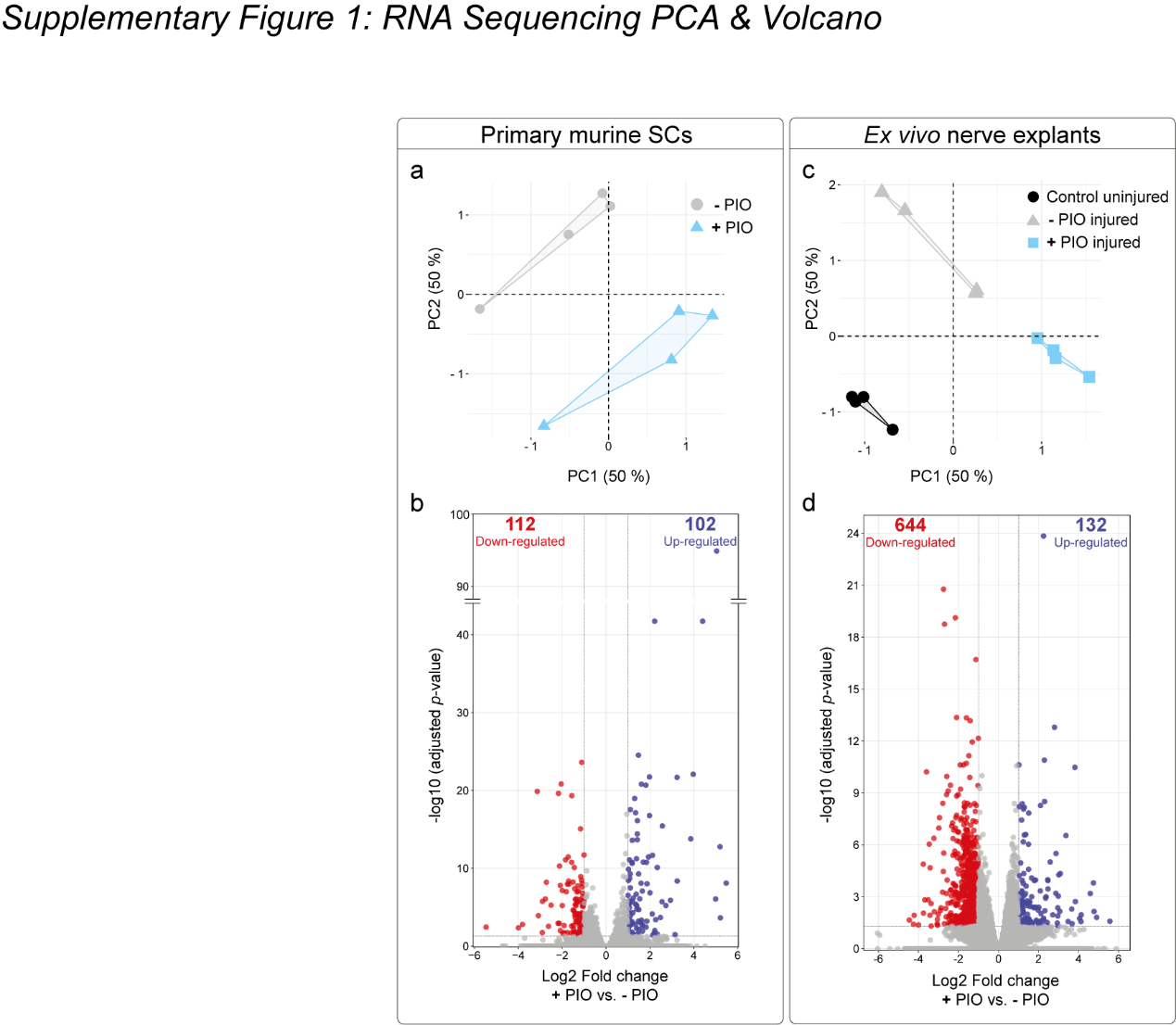


***Supplementary Figure 1:* Differential gene expression in PIO-treated primary SCs and sciatic nerve *ex vivo* explants**

a, b: Bulk RNA sequencing of primary murine SCs treated with either PIO (+ PIO) or DMSO (- PIO) for 48 h. a: Principal component analysis showing distinct clustering of SCs treated with DMSO (- PIO, gray) or PIO (+ PIO, blue). b: Volcano plot of differentially expressed genes in primary SCs comparing + PIO vs. - PIO treatment. Upregulated genes (n= 102) are shown in purple, downregulated genes (n= 112) in red, and non-significant genes (n= 23,483) in gray. c, d: Bulk RNA sequencing of sciatic nerve explants from uninjured control nerves or *ex vivo* injured nerves treated with DMSO (- PIO) or PIO (+ PIO) for 48 h. c: Principal component analysis plot illustrating distinct clustering of uninjured control nerves (black) or injured nerves treated with DMSO (- PIO, gray) or PIO (+ PIO, blue). d: Volcano plot of differentially expressed genes in injured *ex vivo* nerve explants comparing + PIO vs. - PIO treatment. Upregulated genes (n= 132) are shown in purple, downregulated genes (n= 644) in red, and non-significant genes (n= 27,503) in gray. *n*= 4 biological replicates for primary murine SCs and *ex vivo* sciatic nerve explants. Source data are provided as a Source Data file. PC, Principle component; PIO, Pioglitazone; SC, Schwann cell.


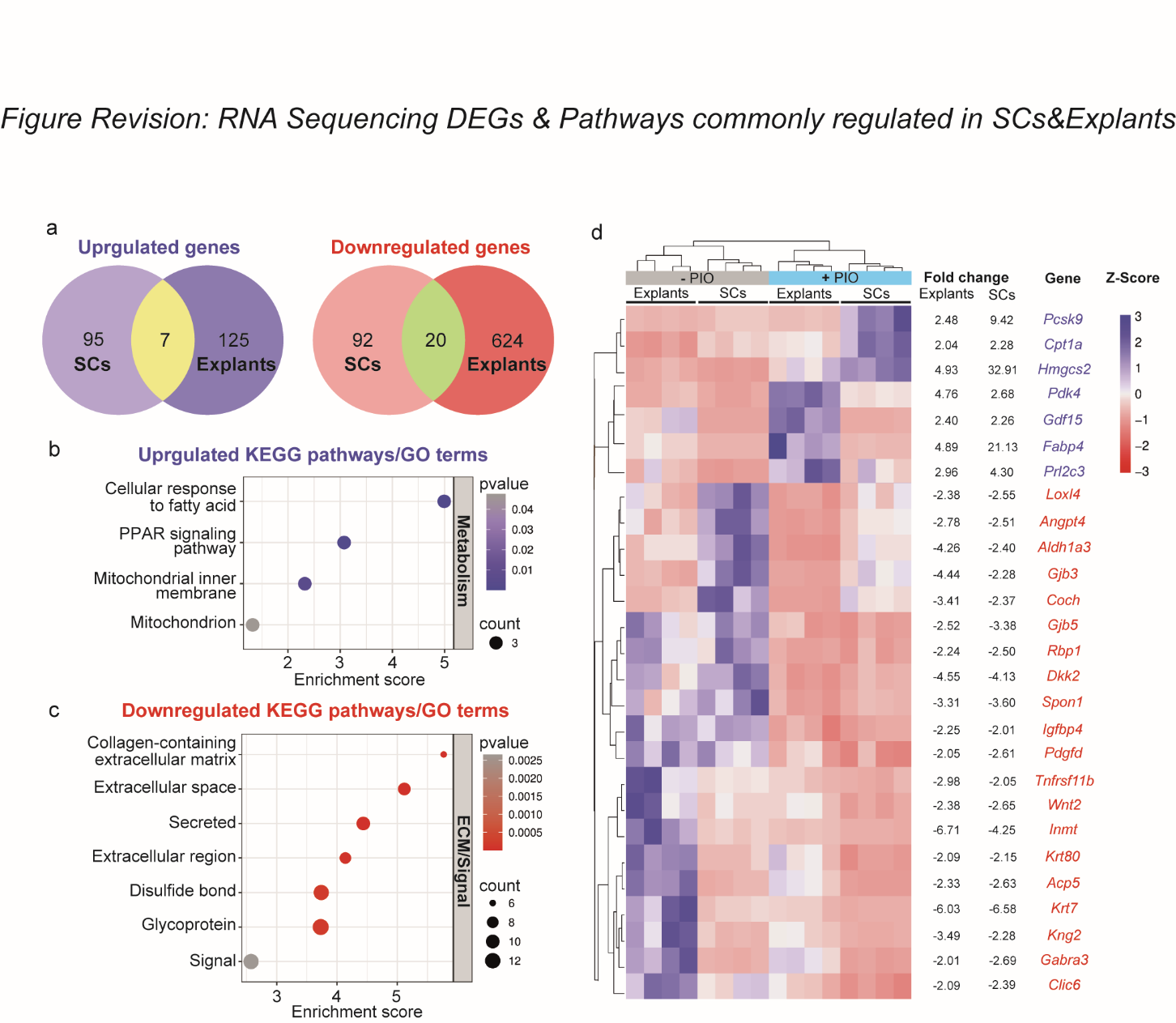


***Supplementary Figure 2:* Shared differential gene and pathway expression in PIO-treated primary SCs and sciatic nerve *ex vivo* explants**

a-d: Bulk RNA sequencing analysis of primary murine SCs and sciatic nerve *ex vivo* explants treated with either PIO (+ PIO) or DMSO (- PIO) for 48 h. a: Venn diagrams depicting the number of shared significantly upregulated and downregulated genes identified in SCs and nerve explants. b, c: Bubble plots representing enriched KEGG pathways and GO terms associated with shared upregulated (b) and downregulated (c) genes between the two experimental systems. d: Heat map illustrating the most significantly differentially expressed genes contributing to the KEGG pathways/GO terms shown in (b) and (c). Shared upregulated genes (n= 7) are indicated in purple, and shared downregulated genes (n= 20) are shown in red. *n*= 4 biological replicates for primary murine SCs and *ex vivo* sciatic nerve explants. GO, Gene ontology; KEGG, Kyoto Encyclopedia of Genes and Genomes; PIO, Pioglitazone; SC, Schwann cell.


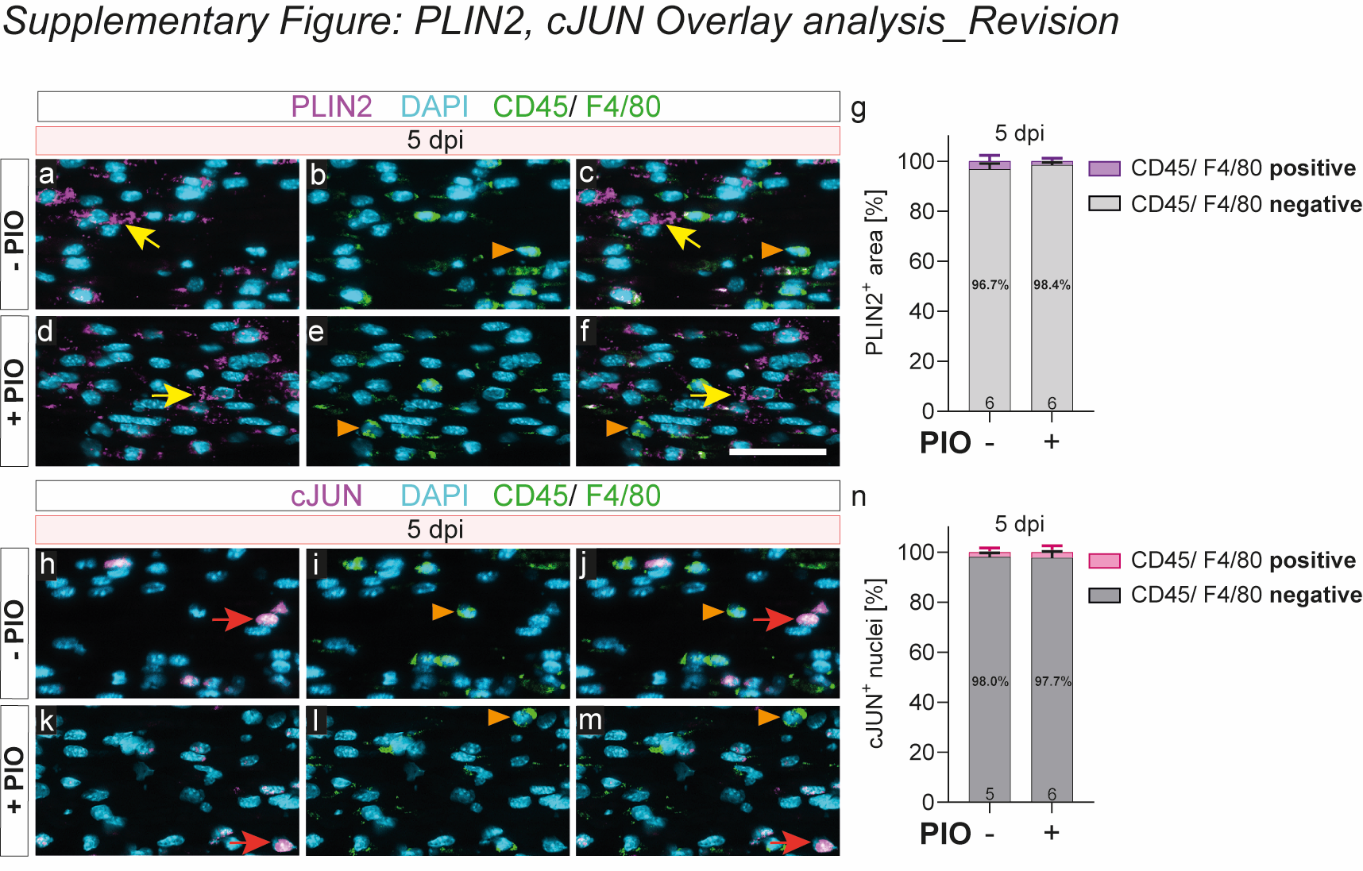


***Supplementary Figure 3:* Analysis of lipid droplet- and repair SC marker expression in immune cells**

a-f, h-m: Histological analysis of longitudinal distal sciatic nerve sections from mice at 5 days post injury. Mice were treated for 5 consecutive days with either PIO (+ PIO) or DMSO (- PIO; vehicle control), starting on the day of injury. Sections were co-stained for PLIN2 (lipid droplet marker; indicated by yellow arrows in a, c, d, f) and CD45/ F4/80 (immune cell markers; indicated by orange arrowheads in b-c, e-f, i-j, l-m) (a-f), or for cJUN (repair SC marker; indicated by red arrows in h, j, k, m) and CD45/ F4/80 (h-m). g: Quantification of the PLIN2^+^ area in CD45/ F4/80 positive immune cells (purple) and in CD45/ F4/80 negative cells (gray) at 5 dpi comparing – PIO and + PIO treatment; *n*= 6. n: Quantification of cJUN^+^ nuclei in CD45/ F4/80 positive immune cells (pink) and in CD45/ F4/80 negative cells (gray); *n*= 5-6. Each dot represents a single mouse. Statistical significance was determined using a two-sided Mann–Whitney test. Error bars represent mean with SD. Significance levels are indicated by asterisks (*P≤ –0.05, **P≤ 0.005, ***P≤ 0.001). Scale bar in f: 50 µm (applies to all images; a-f, h-m). DAPI, 4′,6-Diamidin-2-phenylindol; dpi, days post injury; PIO, Pioglitazone; PLIN2, Perilipin 2; SC, Schwann cell.


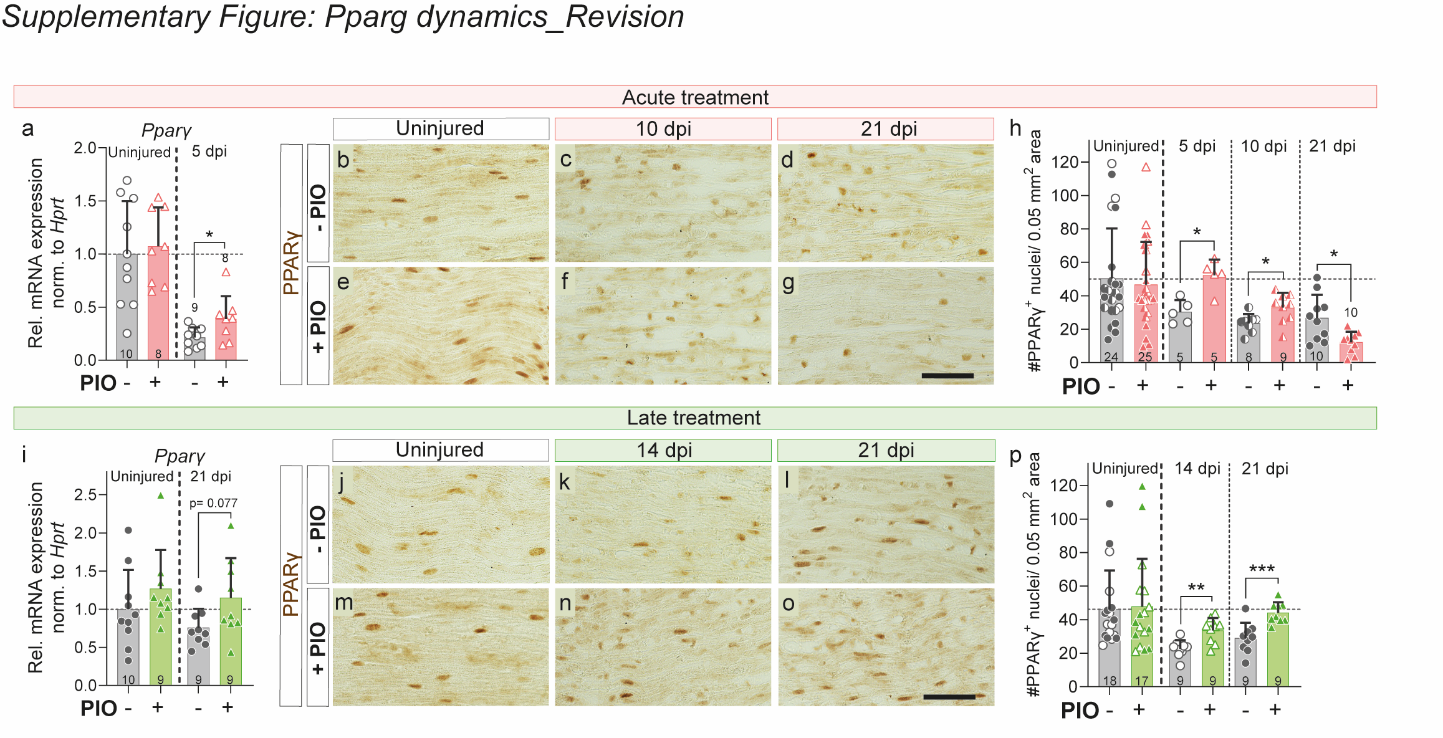


***Supplementary Figure 4:* PPARɣ dynamics in sciatic nerves over the time of regeneration at the mRNA and protein levels­­**

a: qPCR analysis of gene expression of nuclear-encoded *Pparɣ* in distal sciatic nerves in mice receiving “acute” PIO treatment (days 0-5 post injury daily) at 5 dpi and in uninjured control nerves. Gene expression in uninjured samples was normalized to one and fold changes were calculated for PIO-treated samples. *n*= 8-10 as indicated in the graphs. b-g: Immunohistochemical analysis of distal longitudinal sections of murine sciatic nerves stained for PPARɣ during regeneration at 5, 10 and 21 days post injury and in uninjured control nerves. h: Quantification of PPARɣ^+^ nuclei at 5, 10, and 21 dpi and in uninjured nerves (pooled across timepoints) for “acute“ treatment. i: qPCR analysis of gene expression of nuclear-encoded *Pparɣ* in distal sciatic nerves in mice receiving “late” PIO treatment (days 5-21 post injury every second day) at 21 dpi and in uninjured control nerves. Gene expression in uninjured samples was normalized to one and fold changes were calculated for PIO-treated samples; *n*= 9-10 as indicated in the graphs. j-o: Immunohistochemical analysis of distal longitudinal sections of murine sciatic nerves stained for PPARɣ during regeneration at 14, and 21 days post injury and in uninjured control nerves. p: Quantification of PPARɣ^+^ nuclei at 14, and 21 dpi and in uninjured control nerves for “late“ treatment; *n*= 9 as indicated in the graphs. Scale bars in (g, o) represent 50 µm (apply to all images, b-g, j-o). Each dot represents a single mouse. Statistical significance was determined using a two-sided Mann–Whitney test. Error bars represent mean with SD. Significance levels are indicated by asterisks (*P≤ 0.05, **P≤ 0.005, ***P≤ 0.001). dpi, days post injury; PIO, Pioglitazone.


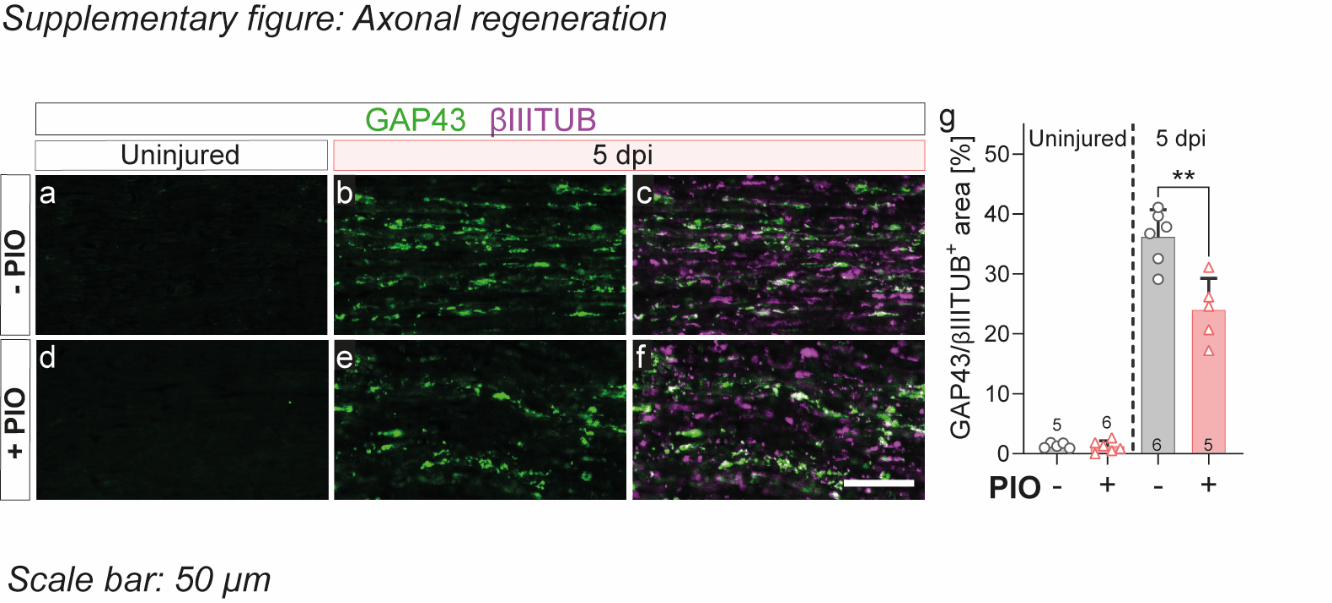


***Supplementary Figure 5:* Acute PIO treatment impairs axonal regeneration following nerve injury**

a-f: Histological analysis of longitudinal sections of murine sciatic nerves stained for the pro-regenerative marker GAP43 at 5 days post injury and in uninjured nerves. Images of injured nerves were taken distal to the lesion site. Mice were treated for 5 consecutive days either with PIO (+ PIO) or DMSO (- PIO; vehicle control), starting on the day of injury. g: Quantification of GAP43/βIIITUB colocalization at 5 dpi and in uninjured nerves; *n*= 5-6 as indicated in the graph. Scale bar in (f) represents 50 µm (applies to all images, (a-f)). Each dot represents a single mouse. Statistical significance was determined using a two-sided Mann–Whitney test. Error bars represent mean with SD. Significance levels are indicated by asterisks (*P≤ 0.05, **P≤ 0.005, ***P≤ 0.001). dpi, days post injury; PIO, Pioglitazone.


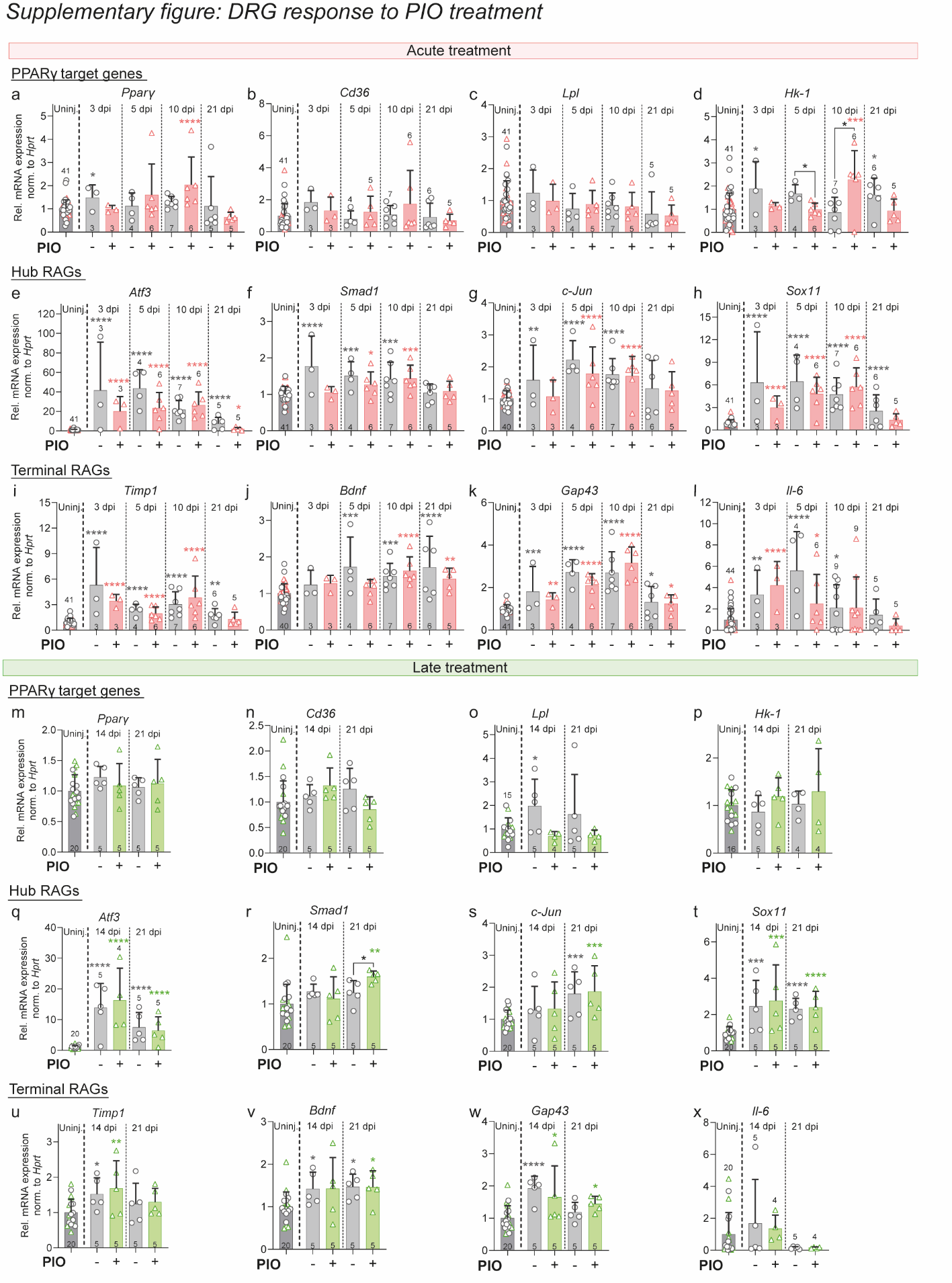


***Supplementary figure 6:* Gene expression analysis in dorsal root ganglion neurons**

a-x: qPCR analysis of gene expression in dorsal root ganglia (DRGs) over the time of regeneration in mice receiving either “acute” (a-l) or “late” (m-x) PIO treatment (acute: 0-5 dpi daily; late: 5-21 dpi every second day). Only DRGs from L3, L4 and L5 from the control and injured side were used in this analysis, since those correspond to the neuronal cell bodies of the sciatic nerve. Gene expression in uninjured samples was normalized to one and pooled across time points and treatments, since no differences could be detected. Fold changes in relation to control DRGs were calculated for other timepoints and treatments. (a-l): Relative mRNA expression levels of PPARɣ target genes, hub RAGs, and terminal RAGs as indicated in mice receiving acute PIO treatment. (m-x): Relative mRNA expression levels of PPARɣ target genes, hub RAGs, and terminal RAGs as indicated for mice receiving late treatment; *n*= 3-9 as indicated in the graphs. Each dot represents a single mouse. Statistical significance was determined using a two-tailed unpaired T-test. All error bars show mean with SD. Statistical significance is indicated by asterisks: When ‘*’ is colored (gray, green or red) significance is compared to uninjured control DRGs. Black ‘*’ indicates significance between treatments within a single timepoint. Significance levels: (* P<0.05, ** P<0.005, *** P<0.001, **** P<0.0001). Uninj., uninjured; dpi, days post injury; PIO, Pioglitazone; RAG, regeneration associated gene.


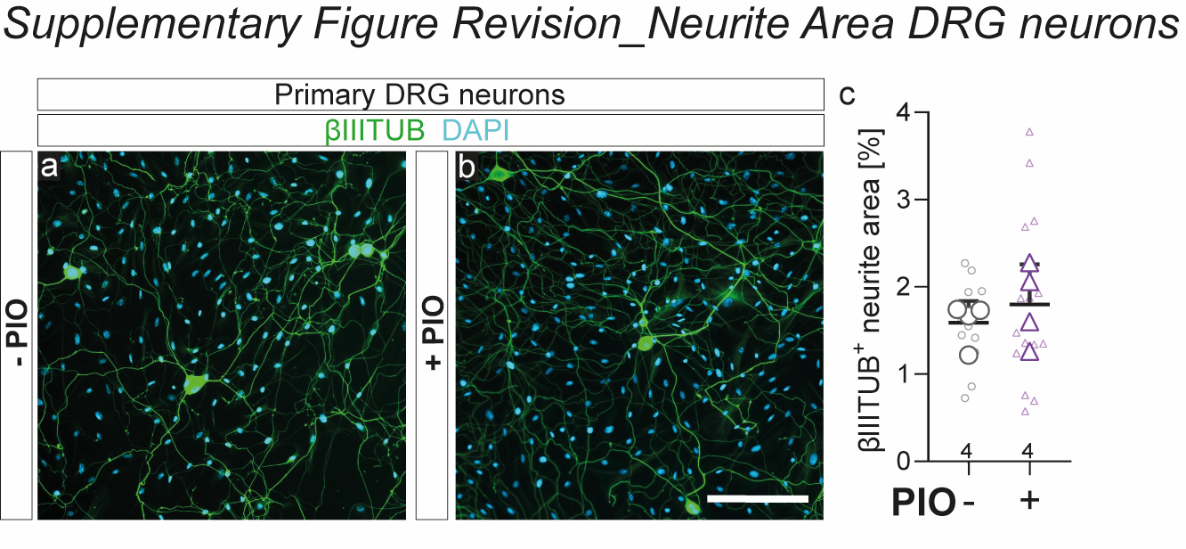


***Supplementary figure 7:* Pioglitazone treatment does not affect neurite outgrowth in primary DRG neurons**

a, b: Primary DRG neurons treated with PIO (+ PIO) or DMSO (- PIO) for 5 consecutive days and stained for the axonal marker βIIITUB. c: Quantification of βIIITUB^+^ neurite area per DRG neuron. Each large dot in (c) represents the mean value from one biological replicate (one mouse), with four technical measurements per replicate shown as smaller background dots. Biological replicates analyzed: *n*= 4. All error bars represent mean with SD. Statistical significance was determined using a two-sided T-test. Statistical significance is indicated by asterisks (* P< 0.05, ** P< 0.005, *** P< 0.001). Scale bar in (b) is 200 µm (applies to a, b). DRG, dorsal root ganglion; PIO, Pioglitazone.


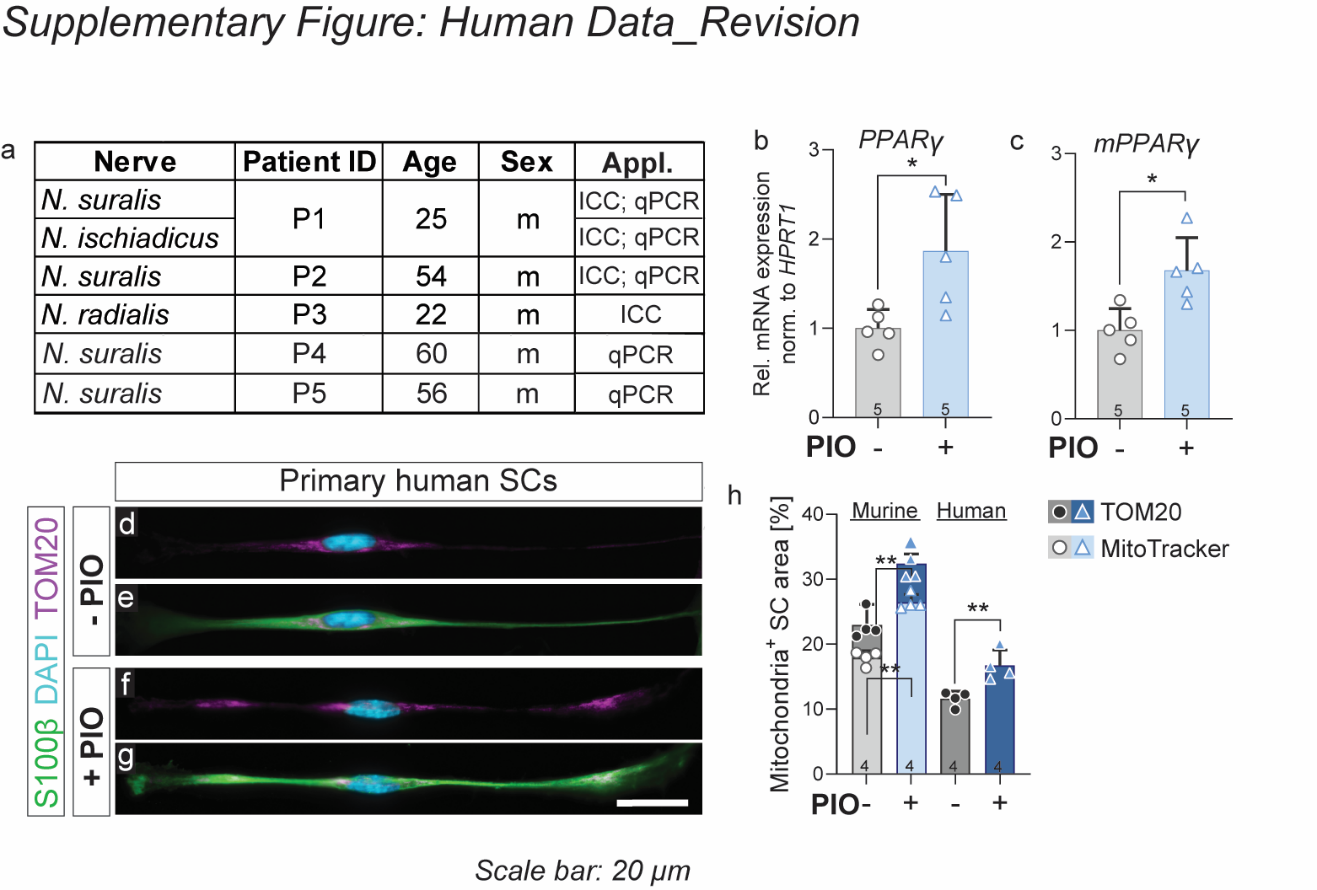


***Supplementary figure 8:* Translational potential of Pioglitazone in primary human SCs: PIO upregulates *PPARɣ* expression and increases intracellular mitochondrial content**

a: Overview of human nerve samples used for assessing PIO responsiveness, including nerve type, patient ID, age, sex, and application method. b-c: qPCR analysis of genes involved in lipid anabolism in primary human SCs treated with either PIO (+ PIO, blue) or DMSO (- PIO, gray) for 5 consecutive days. Gene expression in DMSO-treated human SCs was normalized to one, and fold changes were calculated for PIO-treated samples. d-g: Primary human SCs treated with PIO (+ P­­IO) or DMSO (- PIO) for 5 consecutive days stained for SC marker (S100β) and mitochondria (TOM20). h: Quantification of intracellular mitochondrial content in primary murine (MitoTracker^+^ and TOM20^+^) and human (TOM20^+^) SCs following PIO treatment. Biological replicates analyzed: *n*= 5 in (b-c), *n*= 4 in (h). Each dot represents primary SCs isolated from a single nerve of a human donor (b-c, h), or an individual mouse (h). All error bars represent mean with SD. Statistical significance was determined using a two-sided Mann–Whitney test in (b-c) and a two-sided T-test in (h). Statistical significance is indicated by asterisks (* P< 0.05, ** P< 0.005, *** P< 0.001). Scale bar in (g) is 20 µm (applies to d-g). Appl., application; dpi, days post injury; ICC, immunocytochemistry, m, male; *N*, *Nervus*; PIO, Pioglitazone; qPCR, quantitative Polymerase Chain Reaction; SC, Schwann cell.

*Supplementary Data set 1*

**List of all differentially regulated genes identified by bulk RNA sequencing**

Differentially expressed genes in primary murine SCs treated either with PIO (+ PIO) or DMSO (- PIO) for 48 h.

*Supplementary Data set 2*

**List of all differentially regulated genes identified by bulk RNA sequencing**

Differentially expressed genes in *ex vivo* sciatic nerve explants from uninjured control nerves or injured nerves treated with PIO (+ PIO) or DMSO (- PIO) for 48 h.

**Supplementary methods**

*Supplementary material for Histology*

Primary antibodies: anti-F4/80 (rat, 1:500, Novus biologicals, NB600-404); anti-PPARɣ (rabbit, 1:50, Invitrogen, MA5-14889); anti-GAP43 (rabbit, 1:1000, Millipore, AB5220); anti-βIIITUB (mouse, 1:3000, Covance, MMS435P-200); anti-TOM20 (rabbit, 1:500, Cell signaling, 42406S). Secondary antibodies: goat anti-Rat IgG (H+L) Highly Cross-Adsorbed Secondary Antibody, Alexa Fluor™ 546 (1:500, Invitrogen, A-11081); biotinylated goat anti-Rabbit IgG (H+L) Antibody (1:500, Vector Laboratories, BA-1000); donkey anti-Rabbit IgG (H+L) Highly Cross-Adsorbed Secondary Antibody, Alexa Fluor™ 488 (1:500, Invitrogen, A-21206); goat anti-Mouse IgG (H+L) Cross-Adsorbed Secondary Antibody, Alexa Fluor™ 546 (1:500, Invitrogen, A-11003); goat anti-mouse IgG (H+L) Highly Cross-Adsorbed Secondary Antibody, Alexa Fluor™ 488 (1:500, Invitrogen, A-11001); goat anti-rabbit IgG (H+L) Highly Cross-Adsorbed Secondary Antibody, Alexa Fluor™ 546 (1:500, Invitrogen, A-11010).

A SignalStain Boost detection reagent (Cell signaling, 8114P) was used to detect rabbit primary antibodies in immunohistochemical sections, thereby enhancing the signal intensity of the PPARɣ antibody (rabbit, Invitrogen, MA5-14889). The SignalStain Boost reagent was applied according to the manufacturer’s instructions.

*Imaging and Quantification*

Histological nerve sections were imaged using a Keyence BZ-X810 fluorescence microscope with a 20x objective. One section per animal, either from uninjured nerves or approximately 5 mm distal to the lesion site, was analyzed. Each nerve area was outlined using the ‘segmented line’ tool, and a functional color threshold was applied. The number of PPARɣ^+^ nuclei was quantified using the automated ‘analyze particles’ function in ImageJ. The colocalization of GAP43 and βIIITUB in regenerating axons was evaluated using the ‘selection and overlay’ analysis tool in ImageJ.

Primary DRG neurons were imaged using a Zeiss Axio Observer fluorescence microscope with a 10x objective. Neurite network analysis was performed on four randomly selected images per quadrant for each biological replicate. The total image area (~1.57 mm^2^) was analyzed by applying a functional color threshold, followed by automated quantification using the ‘analyze particles’ function in ImageJ.

Primary human SCs were imaged using a Zeiss Axio Observer fluorescence microscope with a 63x oil immersion objective. Mitochondrial analysis was performed on 30 individual SCs per nerve donor sample. Human SCs were manually outlined using the ‘segmented line’ tool in ImageJ. A standardized color threshold was applied, and the TOM20^+^ area (relative to the S100β signal) was quantified using the automated ‘analyze particles’ function in ImageJ.

*Patient material and sample preparation*

Human nerve tissue was derived from peripheral nerve transplantation surgeries performed at the Department of Neurosurgery, section of peripheral nerve repair at the district hospital of Günzburg (Ulm University, Germany). Written informed consent was given by all patients before surgery. Nerve tissue was kept in Ringer solution until it reached our laboratory. Then, nerve fascicles were separated from surrounding connective tissue, placed in culture media (DMEM/ 10% FCS/ 1%Pen/Strep) and kept for 8-10 days with media change thrice weekly. SCs were then isolated as described for murine nerves in the Materials and Methods section of the main manuscript.

Experiments were approved by the local ethical review board (registration Nr. 261/20 and 224/22).

*List of primers used in the study*

***Supplementary table 1:*** Primer pairs used for qPCR analysis.

| Gene name | Sequence forward primer (5' > 3') | Sequence reverse primer (3' > 5') |
| --- | --- | --- |
| *Acaca* | GCG TCG GGT AGA TCC AGT T | CTC AGT GGG GCT TAG CTC TG |
| *Acadm* | CCA GAG AGC AGC CTG GGA GGT T | CCT CCG AAA ATC TGC ACG GCG T |
| *Atf3* | GCT GGA GTC AGT TAC CGT CAA | CGC CTC CTT TTC CTC TCA T |
| *Bdnf* | ACC ATA AGG ACG CGG ACT TG | GAG TAG AGG AGG CTC CAA AGG C |
| *Cd36* | CCC CGA GGA CCA CAC TGT GTC T | AGG TGG AAA GGA GGC TGC GTC T |
| *c-Jun* | ACC CCC ACT CAG TTC TTG TG | AGT TGC TGA GGT TGG CGT AG |
| *Fasn* | GCT GCT GTT GGA AGT CAG C | AGT GTT CGT TCC TCG GAG TG |
| *Gap43* | CAG GAA AGA TCC CAA GTC CA | GAA CGG AAC ATT GCA CAC AC |
| *Hk-1* | ACA TCG TGC ACG GCA GTG GAA G | ACA TCG TGC ACG GCA GTG GAA G |
| *Hprt* | GGA GCG GTA GCA CCT CCT | CCT GGT TCA TCA TCG CTA ATC |
| *HPRT1 human* | TGA CAC TGG CAA AAC AAT GCA | GGT CCT TTT CAC CAG CAA GCT |
| *Il-6* | GCT ACC AAA CTG GAT ATA ATC AGG A | CCA GGT AGC TAT GGT ACT CCA GAA |
| *Ldha* | TTG GCG GAT GAG CTT GCC CTT G | AGG AAG AGG CTG CCA TGC TGG A |
| *Lpl* | CCC CCA GTC GCC TTT CTC CTG A | AGC TGG TCC ACG TCT CCG AGT C |
| *mPparɣ* | GAC CAC TCG CAT TCC TTT GAC ATC AAG CC | TGA TCG CAC TTT GGT ATT CTT GGA GCT TCA G |
| *Mt. 16S rRNA* | CCG CAA GGG AAA GAT GAA AGA C | TCG TTT GGT TTC GGG GTT TC |
| *Pfka* | ACT CAG GCT GCG TTC TGG GGA T | GCC TCA GCT TCA GCC ACC ACT G |
| *Pparɣ* | GAA AGA CAA CGG ACA AAT CAC C | GGG GGT GAT ATG TTT GAA CTT G |
| *Smad1* | GGT CTG CAT CAA CCC CTA CC | GAA CTG AGC CAG AAG GCT GT |
| *Sox11* | GAG CTG AGC GAG ATG ATC G | GAA CAC CAG GTC GGA GAA GT |
| *Timp1* | GCA AAG AGC TTT CTC AAA GAC C | AGG GAT AGA TAA ACA GGG AAA CAC T |
